# Boundaries of eliminated heterochromatin of *Tetrahymena* are positioned by the DNA-binding protein Ltl1

**DOI:** 10.1093/nar/gkz504

**Published:** 2019-06-13

**Authors:** Vita N Jaspan, Marta E Taye, Christine M Carle, Joyce J Chung, Douglas L Chalker

**Affiliations:** Biology Department, Washington University in St. Louis, St. Louis, MO 63130, USA

## Abstract

During differentiation of the *Tetrahymena thermophila* somatic nucleus, its germline-derived DNA undergoes extensive reorganization including the removal of ∼50 Mb from thousands of loci called internal eliminated sequences (IESs). IES-associated chromatin is methylated on lysines 9 and 27 of histone H3, marking newly formed heterochromatin for elimination. To ensure that this reorganized genome maintains essential coding and regulatory sequences, the boundaries of IESs must be accurately defined. In this study, we show that the developmentally expressed protein encoded by *L*ia*3*-*L*ike *1* (*LTL1)* (Ttherm_00499370) is necessary to direct the excision boundaries of particular IESs. In *ΔLTL1* cells, boundaries of eliminated loci are aberrant and heterogeneous. The IESs regulated by Ltl1 are distinct from those regulated by the guanine-quadruplex binding Lia3 protein. Ltl1 has a general affinity for double stranded DNA (*K*_d_ ∼ 350 nM) and binds specifically to a 50 bp A+T rich sequence flanking each side of the D IES (*K*_d_ ∼ 43 nM). Together these data reveal that Ltl1 and Lia3 control different subsets of IESs and that their mechanisms for flanking sequence recognition are distinct.

## INTRODUCTION

The organization of DNA within the nucleus reflects how chromosomes are partitioned into functional domains. The widening use of chromosome conformation capture (3C)-based studies has illuminated the extent to which chromosomal organization is correlated with gene expression during differentiation (1) and even exhibits conservation between species (2,3). The existence of observable domains requires that eukaryotes, either actively or passively, create and maintain boundaries between individual genomic regions. Even so, we have limited knowledge of the spectrum of mechanisms that ensure separate chromosomal domains, and most of our conceptual framework comes from studies of a small number of proteins that act to maintain boundaries.

The ciliate *Tetrahymena thermophila*—which has a somatic macronucleus and a germ line micronucleus—offers an ideal model in which to study how heterochromatin is established and is partitioned into distinct chromatin domains. During development of its somatic genome, *Tetrahymena* packages ∼12 000 loci (totaling ∼1/3 of the 157 Mb genome) dispersed throughout the germline-derived genome into heterochromatin (4). These cells use small RNAs to identify these loci and target methylation of the associated chromatin on lysines (K) 9 and 27 of histone H3 (5–7). However, whereas other eukaryotes stop at this point, *Tetrahymena* takes the process one step further and eliminates this heterochromatin along with the associated sequences, thereby creating a streamlined somatic genome that supports efficient gene expression (8). The advantage *Tetrahymena* thus offers over other models is that, because all heterochromatic loci are excised from the somatic genome, we can unambiguously identify all loci that are targets for heterochromatin formation during development. Furthermore, the boundaries of these heterochromatic sequences can be defined by the sites of excision.

The individual loci that are targets for heterochromatin formation and subsequent excision from the developing genome are called internal eliminated sequences (IESs). The ∼12 000 IESs are unevenly distributed throughout the *Tetrahymena* genome, with higher density near the center of germline chromosomes and lower density in the arms (4). They can range in size from a few hundred base pairs (bp) to >20 kb. Many IESs are largely composed of unique sequences without easily identifiable features, whereas others contain repetitive sequences and/or are derived from transposable elements (9–13). Given that the eliminated loci are interspersed and in close proximity to genes, the boundaries between the eliminated heterochromatin and the rest of the genome must be accurately positioned to prevent loss of functional sequences.

Even though the excision boundaries of the thousands of IESs can be mapped simply by comparing the micronuclear (intact) and macronuclear (rearranged) genome sequences, few features or sequence motifs are apparent, which provides little insight as to how the cell can accurately and efficiently eliminate a third of its genome. Mutational analyses of IESs consistently revealed that IES boundaries are regulated by *cis*-acting sequences in the flanking regions to the IESs, while the scnRNAs recognized the eliminated regions (14–19). For example, Godiska and Yao demonstrated the importance of polypurine tracts flanking the M IES (16). They found that these flanking sequences, located ∼45 bp away from the IES boundaries, were necessary for accurate excision of the IES. Similarly, Chalker *et al.* identified a 70 bp *cis*-acting region flanking the R IES that is essential for its accurate elimination (14). When *cis*-acting sequences were moved, the IES boundaries also moved, such that they remained a conserved distance away from these *cis-*acting sequences. Though these *cis*-acting sequences were shown to be important for accurate determination of IES boundaries, there is no consistent sequence motif found across the flanking regions of all IESs. Thus, there is no obvious universal mechanism to recognize and control excision boundaries of all IESs.

Some IESs, including the well-studied M IES, are flanked by polypurine tracts (5′-A_5_G_5_ -3′) that are recognized by the Lia3 protein (20). In the absence of Lia3, boundaries of these IESs are aberrant and heterogeneous. These polypurine tracts were shown to form guanine (G)-quadruplex structures, which Lia3 binds with high affinity (20). However, only a few hundred of the thousands of IESs have polypurine tracts and are regulated by Lia3 (21). Most of the IESs within the *Tetrahymena* genome lack terminal repeats or any other obvious flanking sequence motifs. For this reason, we hypothesized that other proteins must play a role in the control of the excision of these IESs.

Lia3 was identified as a candidate regulator of IES excision due to its localization in developing macronuclei at the time that IESs are removed from the genome. The *Tetrahymena* genome encodes three Lia3-like paralogs, all which share similar expression patterns (20). In this study, we investigated the possibility that the Lia3 paralog, Lia3-like 1 (encoded by *LTL1* -Ttherm_00499370) controls IES excision boundaries. Ltl1 shares similarity with Lia3 across its central 100 amino acid long region and, consistent with a role in DNA elimination, localizes to the developing macronuclei (20). Cells lacking *LTL1* produced viable progeny, but failed to accurately position excision boundaries for ∼18% of the IESs tested, all of which are distinct from those previously found to be regulated by Lia3. Despite structural and functional similarities to Lia3, we found that Ltl1 binds preferentially to a long (50bp) A+T rich regulatory region, not a G-quadruplex. We propose that differential binding of distinct boundary regulators is critical to ensure that individual heterochromatin domains are accurately partitioned prior to DNA elimination.

## MATERIALS AND METHODS

### Cell stocks and maintenance

*Tetrahymena* cell lines were grown and maintained in either 1× SPP (1% proteose peptone, 2% dextrose, 0.1% yeast extract, and 10μM FeCl_3_) or 1× Neff's medium (0.25% proteose peptone, 0.25% yeast extract, 0.5% dextrose and 10 μM FeCl_3_) at 24–30°C. Penicillin/streptomycin (250 μg/ml each) and Amphotericin B (1.25 μg/ml) was regularly added to cultures to prevent contamination. Wild-type inbred *Tetrahymena* strains CU428 [*mpr1–1/mpr1–1* (*MPR1*; mp-s, VII)], B2086 (II), and CU427 [*Chx1–1/Chx1–1* (VI, cy-s)] were used to generate mutant strains and transformed lines. Star strains B*(VI) and B*(VII) were mated with heterozygous germline knockout lines to generate homozygous cell lines. Cells were made competent to mate by removal from growth medium by centrifugation at ∼1100 × g, washing cells with 10 mM Tris–HCl (pH 7.5), and allowing them to starved overnight. To initiate mating, equal numbers of starved cells of two different mating types were mixed and incubated in dishes at 30°C without agitation for at least two hours. To assess progeny viability, mating pairs were isolated into ∼30 μl drops of 1× SPP, then replica plated to medium containing antibiotics. To monitor progression through mating, cells were fixed with 2% paraformaldehyde, stained with 1μl of DAPI (10 μg/μl), and visualized by fluorescence microscopy. Fixed cells were observed under 60× oil immersion lenses of a fluorescent Nikon E600 microscope.

### DNA amplification

Polymerase chain reactions (PCR) were performed using either *Phusion* or *Taq* DNA polymerases. *Phusion* reactions contained 1× Phusion-HF PCR buffer (New England Biolabs, Ipswich, MA, USA), 1.5 to 3.5 mM MgCl_2_, 0.25 μM of each oligonucleotide, 125 μM dNTPs, and NEB Phusion DNA polymerase (New England BioLabs, Ipswich, MA, USA). Amplification reactions using *Taq* DNA polymerase contained 1× GoTaq PCR buffer, 2.5 mM MgCl_2_, 0.25 μM of each oligo, 0.1 mM dNTPs, and *Taq* DNA Polymerase. Routinely, the following PCR cycling conditions were used: 94°C for 3 min, then 25 to 30 cycles of 94°C for 30 s, 53–56°C for 30 s, 72°C for 60 s/kb of product, followed by a final extension at 72°C for 5 min. Oligonucleotide primers were designed with the assistance of Primer3 (22,23) and synthesized by Integrated DNA Technologies (IDT, Coralville, IA, USA); sequences used in this study are provided in Supplementary Tables S1 and S2.

PCR products analyzed by fractionation on 1–1.5% agarose/1× TBE (Trizma Base, boric acid, 0.25 M EDTA pH 8.0) gels containing 0.2μg/ml ethidium bromide. The co-migration of GeneRuler 100 bp or 1 kb ladders (ThermoFisher Scientific, Waltham, MA, USA) were used to estimate sizes of PCR products.

### Creation of LTL1 homozygous germline knockout strains

The entire coding region of *LTL1*, encompassing from 17 bp upstream of the start codon to 2 bp beyond the stop codon, was deleted from both the micro- and macronuclear genomes, replaced with the *NEO3* (*MTT1-NEO*) selection cassette (24). The plasmid created to disrupt *LTL1* contained 1073 bp corresponding to the genomic region immediately upstream and 1009 bp downstream of the *LTL1* coding region. These DNA segments were amplified by PCR with oligonucleotide primer pairs 499_KOup_FW and 499_KOup_RV or 499_KOds_FW and 499_KOds_RV, respectively (Supplementary Tables S1). Amplified products were cloned into pCR2.1 by topoisomerase-mediated cloning (Topo-TA cloning kit, Life Technologies). The cloned downstream flanking DNA was removed from pCR2.1 by digestion with restriction enzyme *Kpn*I and inserted into the *Kpn*I site in the plasmid containing the upstream flanking region to create pCR2.1_LTL1flanksA-J. The *NEO3* selectable marker was removed from pENTR-*NEO3* (25) by digestion with *Bsr*GI and *Asc*I and cloned pCR2.1_LTL1flanksA-J digested with *Bsi*WI and *Asc*I to generated *LTL1-NEO3* knockout construct.

The *LTL1-NEO3* knockout construct was introduced into mating *Tetrahymena* WT strains CU428 and B2086 using biolistic transformation (26). Between 2h15m and 3 h post-mixing, 1 × 10^7^ mating pairs were harvested by centrifugation for two minutes at 1000 × g, distributed onto Whatman 50 filters pre-moistened with 10 mM Tris–HCl and then bombarded with gold microcarriers coated with 1 μg of *LTL1-NEO3* linearized plasmid by using a BioRad PDS 1000 He gene gun assembled with a single 900 psi rupture disc. After each particle bombardment, cells were then transferred to 25 mls of 10 mM Tris–HCl and incubated at 30°C overnight to allow mating to complete. Induction of *NEO3* expression was induced by addition of 25 mls of 2× SPP containing 1 μg/ml CdCl_2_. After 4 hours, transformants were selected by addition of CdCl_2_ to 1μg/ml and paramomycin to 80μg/ml.

*LTL1-NEO3* transformed cells surviving paromomycin selection were replated into 1× SPP containing 15 μg/ml 6-methylpurine (6MP) to select for progeny cells. Initial heterozygous transformants were made homozygous by crossing with micronuclear-defective ‘star strains,’ B*VI and B*VI. Without functional micronuclei, star strains are unable to generate viable gametic nuclei at the completion of meiosis, and the mating partner cell donates a haploid micronucleus to the star partner. Cells then abort development and endoduplication creates two cells with identical homozygous micronuclei derived from the non-star cell's micronucleus (27). The ex-conjugants from star crosses were subsequently crossed to wild-type strains to identify cells lines that are homozygous germline knockouts. Homozygous germline knockouts were crossed to produce strains lacking all copies of LTL1 from micro- and macronuclei.

### Genotype analysis of knockout lines

DNA from putative knockout lines was used in PCR assays and Southern blot analysis to confirm disruption of *LTL1*. *Tetrahymena* genomic DNA was isolated by harvesting cells by centrifugation and disrupting the pellet in ∼10 volumes of nuclei lysis solution (Promega, Madison, WI) at 65°C for 30 min, followed by treatment with 30 μg RNAseA at 37°C for 15 min. Denatured protein and cell debris was removed by vigorous mixing with one-third volume of protein precipitate solution (Promega, Madison, WI, USA) followed by centrifugation for 3 min at 13 000 × g; subsequently DNA was recovered by isopropanol precipitation. Genomic DNA was used as template in PCR with three primers to detect the presence of both the *NEO3* knockout allele and the wild-type *LTL1* gene (primers used: p452–3351 and 499_KOds_RV/499370_WT_fw and 499370_WT_rv - a schematic of the primer locations is shown in Figure 2A and sequences are listed in Supplementary Table S2). Southern blot hybridization analysis was performed to ensure that no *LTL1* WT DNA was present in the knockout strains. Genomic DNA was digested with FastDigest *Xba*I (Fermentas) and then fractionated by electrophoresis through a 1.2% agarose/0.5× Tris–HCl–borate–EDTA gel at 130 V for ∼2 h. Fractionated DNA was treated with acid depurination solution (0.25 N HCl) followed by base denaturation solution (0.5 N NaOH) and transferred to Magna uncharged membrane overnight using a downward capillary action. The membrane was washed in 2× SSC and the DNA was cross-linked to the membrane using a Bio-Rad GS Gene Linker UV Chamber (150 mJ). The membrane was pre-hybridized in Southern Hybridization Solution (75 ml 20× SSC, 0.1 M Tris pH 7.5, 10 ml 50× Denhardts, 12.5 ml 10% SDS, 127.5 ml ddH_2_O) at 65°C. DNA corresponding to the *LTL1* genomic region was hybridization with a radiolabeled probe; a *Bsr*GI/*Eco*RI fragment isolated from pCR2.1_LTL1flanksA-J. The probe was denatured by heating to 95 degrees Celsius for 2 minutes, added to the membrane, and allowed to hybridize overnight. The membrane was then washed with 0.5× SSC/1% SDS 5 times and exposed onto a phosphor-imager screen overnight.

### Gene expression analysis

Wild-type strains, B2086 and CU428, and *LTL1* knockout lines were cultured in 1× SPP overnight at 30°C. Cells were harvested by centrifugation, washed, and starved in 10 mM Tris–HCl (pH 7.5) overnight. The following day, cell culture densities were measured using a spectrophotometer set at 540 nm. Equal concentrations of cells were mixed and incubated at 30°C. RNA was isolated from each mating after 3, 6, 9 and 12 h of mating, as well as from growing and starving cells. Before isolation of RNA, a portion of cells from culture was fixed with 2% paraformaldehyde, stained with 1 μl of DAPI (10 μg/μl), and visualized by fluorescence microscopy to monitor how the cells were progressing through mating. Fixed cells were observed under 60x oil immersion lenses of a fluorescent Nikon E600 microscope. The remainder of the cells were concentrated by centrifugation and RNA was extracted using RNA-sol as previously described (28). To monitor expression of *LTL1* in WT and knockout lines, reverse transcription (rt)PCR was performed as described (29) using either *LTL1* or *HHP1 (TTHERM_00705240)* -specific primer pairs, respectively. Imaging of Ltl1-CFP expressing cells was performed as described (29).

### Screening of IESs

To determine whether disruption of *LTL1* affected IES excision, PCR was performed to amplify IES containing loci. By comparing the sizes of these PCR products between wild-type and mutant cells both the importance of *LTL1* in the accuracy and efficiency of IES excision could be assessed. Twenty-seven IESs (named VNJ1–VNJ15 and JC1–JC12) were identified by aligning macronuclear and micronuclear *Tetrahymena* genomic DNA sequences surrounding the previously identified M and R IESs to the micronuclear genome and a second ∼100 kb non-linked genomic region. DNA sequences downloaded from the *Tetrahymena* Genome Database (ciliate.org) (30,31). Sequences present in the micronuclear but not macronuclear genome were defined as IESs. Oligonucleotide primers were designed to amplify each IES and between 100 and 400 bp of flanking DNA (see Supplementary Table S3). Primers used to amplify IESs affected by loss of *LIA3* or other loci unlinked to these two regions are described elsewhere (20,32). Genomic DNA isolated from the progeny of WT strains or *ΔLTL1* strains was used as template for PCR using *Taq* polymerase. PCR products were fractionated on 1.4–1.5% agarose gels and imaged.

For IESs found to have alternate or aberrant boundaries in mutant cells, PCR products corresponding to putative aberrant rearrangement events were gel isolated using a Promega Wizard^®^ SV Gel and PCR Clean-Up System (Promega, Madison, WI). Amplified products were cloned into pCR4 by topoisomerase-mediated cloning (Topo-D cloning kit, Life Technologies), and electroporated into *Escherichia coli*. Recombinant plasmids were isolated from kanamycin-resistant *E. coli* by using a ThermoFisher Scientific GeneJET Plasmid Miniprep kit (ThermoFisher Scientific, Waltham, MA), and DNA sequencing reactions were performed with either M13 forward and reverse primers and BigDye terminators (Applied Biosystems). Sequences were then aligned to the WT micronuclear sequence to determine the mutant excision boundaries.

### Protein purification

A codon-optimized *LTL1* construct (*LTL1s*) was designed and synthesized (IDT, Coralville, IA, USA) for expression in *E. coli*. The synthetic gene was amplified using Phusion polymerase with primers LTLs_R and LTLs_L (see Supplementary Table S4) and cloned into pENTR/D. Subsequently a BamHI/HindIII fragment was fuse in frame with the maltose binding protein (MBP) in a pMAL plasmid. The pMAL-*LTL1* plasmid with the desired sequence was transformed into *E. coli* strain BL21(DE3) for expression and protein purification.

To test whether BL21 cells expressed MBP-Ltl1, whole-cell protein extracted from *E. coli* transformants was examined by western blot analysis. Cell pellets were boiled for 10 min in 2× Laemmli lysis buffer + β-mercaptoemethanol (mixed 20:1) and solubilized protein was fractionated on a precast 4–20% gradient SDS-polyacrylamide gels (Bio-Rad, Hercules, CA, USA) at 145 V for ∼45 min. Fractionated proteins were transferred to nitrocellulose membranes by semi-dry electroblotting at 1.5 mA/cm^2^ for ∼1 h. Membranes were then soaked in 1× PBS + 5% milk to block non-specific binding of antibodies. The fusion protein was detected by chemiluminescence using SuperSignal West Dura substrate after first incubating membranes with mouse anti-MBP antibodies (1:5000 dilution in 2.5% milk/1× PBS), followed by addition of horse radish peroxidase conjugated secondary antibody (1:15 000 dilution of goat-anti-mouse antibodies).

The MBP-Ltl1 fusion protein was purified from 500 ml freshly cultured BL21 cells. Cells were grown in Luria broth (LB) at 37°C, with shaking at 200 rpm, until the optical density (O.D.) at 600 nm reached between 0.6 and 0.9. Protein expression was induced by addition of 500 μM IPTG and the culture was incubated at 18°C, shaking at 200 rpm, overnight to allow for protein accumulation. The following day, cells were harvested, washed in ice-cold PBS, and resuspended in 2× pellet volume of column-buffer (80mM Tris pH 8.0, 500mM NaCl) containing 2 mM PMSF and 1× protease inhibitor cocktail (Sigma-Aldrich, St. Louis, MO, USA). Cells were lysed in a French Press at 1200 PSI and cell debris was removed by centrifugation at 30 000 × g for 20 min at 4°C. Amylose resin beads (NEB, Ipswich, MA) equilibrated in column buffer was mixed with the lysate supernatant and the protein was allowed to bind by rotating at 4°C for 90 min. The protein bound-resin was transferred to a poly-prep chromatography column (Bio-Rad, Hercules, CA, USA), washed twice with column buffer, and the protein was eluted with 10 mM maltose in column buffer. Eluted fractions were dialyzed against 100 mM KCl, 10% glycerol, 50 mM Tris pH 7.5, 1 mM MnSO_4_, 1 mM MgCl_2_, 1 mM ZnSO_4_ overnight. Eluted protein was fractionated on 10% SDS PA gels and visualized by staining with coomassie blue.

### Binding assays

Isolated protein was used for Electrophoretic Mobility Shift Assay (EMSA) as described in Carle *et al.* (20). Oligonucleotides used as probes were end-labeled with ATP [γ^32^P] using T4 PNK and included T4 PNK Buffer (NEB), with 0.5 μl of each 100 μM oligonucleotide for 1–2 h at 37°C and purified from unincorporated nucleotides using G25 Spin Column (Roche Diagnostics). Double-stranded probes were prepared by mixing the labeled probe with its unlabeled reverse complement oligonucleotide in 10 mM Tris (pH 7.5)/5% glycerol and 100 mM KCl (sequences shown in Supplementary Table S5).

### Phyre analysis

Phyre^2^ (**P**rotein **H**omology/analog**Y R**ecognition **E**ngine V 2.0) analysis was used to find proteins with structural similarity, even in the absence of sequence similarity (33). The amino acid sequence used was taken from the TGD (Ciliate.org, search: Ttherm_00499370).

### Generation of Lia3/Ltl1 chimera

*LIA3*/*LTL1* chimeric constructs that swapped the central ∼100 amino acid regions of similarity were created by stitching DNA fragments together by PCR using oligonucleotide primers shown in Supplementary Table S1. Chimeric PCR fragments were cloned into pENTR-D. To generate *Tetrahymena* expression constructs, these chimeric coding sequences were recombined into pBSICC-gtw, which fuses CFP and a cadmium inducible *MTT1* promoter to the insert, using the LR Clonase II recombinase (Life Technologies). The resulting pBSICC-*LTL1, LIA3*, and *LTL1/LIA3* chimeras were digested in *Sac*I HF and *Pvu*I-HF and introduced into the macronuclei of *ΔLIA3* or *ΔLTL1* knockout strains by biolistic transformation (34,35). Transformed cells were grown in 1x SPP and selected based on cycloheximide resistance (12.5μg/ml).

To assess the ability of introduced constructs to rescue the mutant phenotype, transformed cell cultures were starved overnight in 10 mM Tris–HCl, then mixed to induce mating. Between 3 and 4 h after mixing cells, CdCl_2_ was added to 0.05 μg/ml to one of two duplicate crosses to induce expression of the introduced CFP fusion protein. Starting 8 h after mixing cells, single mating pairs were isolated into 30 μl droplets of 1xSPP and incubated at 30°C for 2–3 days. Drops containing viable cells were replica plated to fresh 1× SPP and 1× SPP containing cycloheximide (12.5 μg/ml) to identify cycloheximide-sensitive cells, which indicated loss of the transformed macronuclei. Genomic DNA was isolated from these progeny cells and used to assess the accuracy of DNA rearrangement. PCR analysis of the M element locus was used to assess rescue of the *ΔLIA3* phenotype and of the D IES to assess rescue of the *ΔLTL1* phenotype.

## RESULTS

### *LTL1* regulates the excision boundaries of a distinct set of IESs

**Figure 1.**
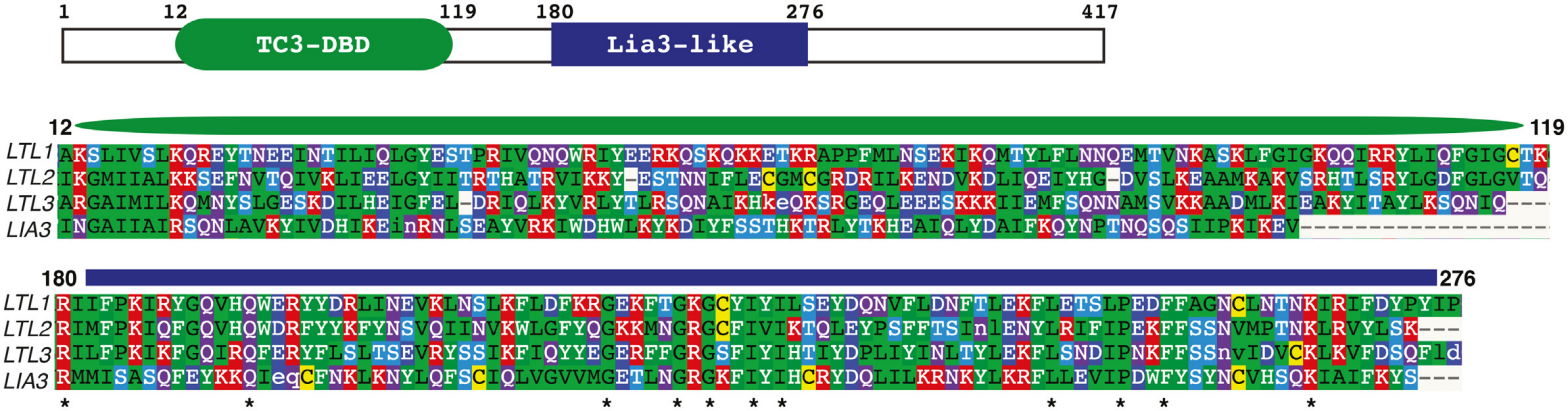
Ltl1 shares similarity with Lia3 and Tc3-related proteins. Schematic depicting the 417 amino acid (aa) coding region and the locations of two recognizable domains: the ∼100 aa region common to Lia3 and two other Lia3-like proteins (blue bar), and an amino terminus with similarity to the helix-turn-helix DNA binding domain found in Tc3 and related transposons (green oval). Asterisks denote aa's conserved across all Lia3-like proteins. Amino acids are colored based on similarity in properties.

We showed previously that Lia3 binds to 5′A5G5 3′-containing guanine quadruplexes and controls the accuracy of excision boundaries of IESs that are flanked by these regulatory sequences. Lia3-regulated loci comprise a few hundred of the thousands of IESs and whether other boundary regulators exist was yet to be discovered. We reasoned that strong candidates to control the boundaries of other IESs include three *L*ia three-like (Ltl) proteins, that share similar developmental expression timing, macronuclear localization, and homology with Lia3 within a central 100 amino acid (aa) domain (Figure 1) (20). Of these, *LTL1* exhibited the highest relative expression (36). In addition, protein structural prediction performed using Phyre2 (33) revealed similarity between the Ltl1 N-terminal region and the bipartite DNA-binding domain of the Tc3 transposase (Figure 1). This model covered 24% of the Ltl1 coding region with 90.8% confidence. Slightly lower confidence models showed similarities to other DNA/RNA binding domains, as well as a mariner transposable element. These features compelled us to investigate its function.

**Figure 2.**
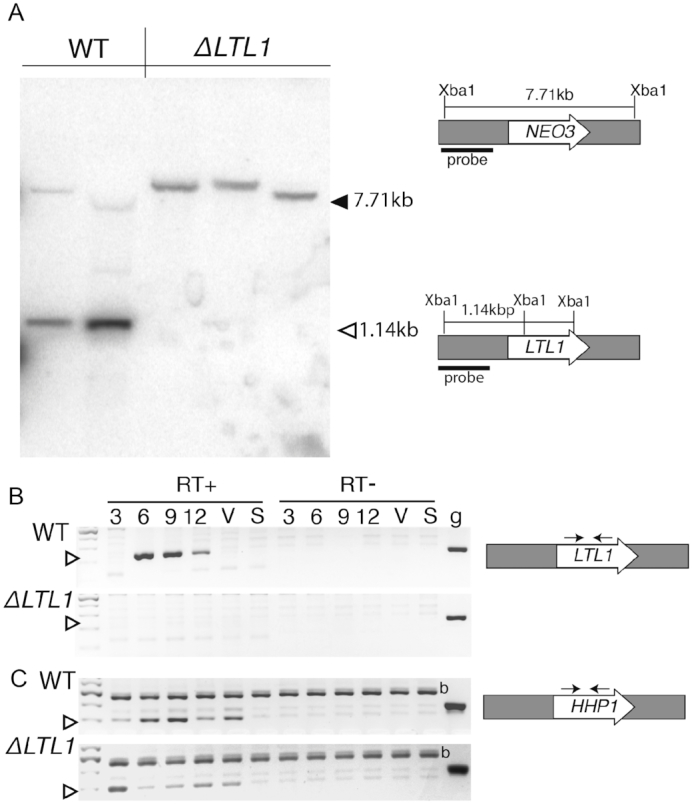
Disruption of LTL1. (**A**) Southern blot analysis of genomic DNA isolated from both wild-type (WT) and *ΔLTL1* after digestion with XbaI. Diagrams of the WT (*LTL1*) and *ΔLTL1* (NEO3) alleles and the expected sizes of each after XbaI digestion are illustrated on the right. The black bar denotes the region corresponding to the radiolabeled probe. The expected positions of migration of WT and *ΔLTL1* fragments are indicated by the solid and open left-facing arrowheads, respectively. (B, C) Reverse transcription PCR analysis of RNA isolated from WT and *ΔLTL1* cells after 3, 6, 9, or 12 h of mating, vegetatively growing (V), or starving (S) cells. PCR was performed with primers amplifying (**B**) *LTL1* or (**C**) the ubiquitously expressed *HHP1* gene and using either cDNA (RT+), RNA w/o cDNA conversion (RT–), or genomic DNA (g). The open arrowhead indicates the expected size of amplified cDNA. *HHP1* primers amplified a non-specific product (b) in these samples with or without cDNA conversion.

To test whether Ltl1 regulates the boundaries of IESs, we generated full (germline and somatic) *LTL1* knockout strains (*ΔLTL1)* by replacing its coding region with the *NEO3* selectable marker (24). Homologous recombination of the *LTL1-NEO3* construct into the *LTL1* locus initially produced heterozygous (*ΔLTL1/+)* strains, which we used in subsequent crosses to generate homozygous *ΔLTL1* strains missing all wild-type (WT) copies (see materials and methods for details). We used PCR-based assays to identify three putative *ΔLTL1* strains (3-1, 4-1 and 5-1) and obtained the expected 1380 bp product when using primers designed to detect the integrated *LTL1-NEO3* construct but observed only non-specific products when using primers designed to amplify the wild-type (WT) gene, indicating complete loss of *LTL1* (Supplementary Figure S1). We then isolated genomic DNA from these strains and used Southern blot analysis to confirm *LTL1* deletion. DNA was digested with *Xba*I, which cuts at sites within the coding region that is removed by insertion the *NEO3* marker. A radiolabeled probe that corresponds to the genomic region immediately upstream of the gene detected the wild-type and knockout alleles as 1147 bp and 7711 bp *Xba*I fragments, respectively (Figure 2A). The wild-type sized fragment was absent from all knockout lines, confirming deletion of *LTL1* (Figure 2A). Because the probe sequence spans an *Xba*I site upstream of the *LTL1* gene, we also detected a faint larger-sized band in the WT sample corresponding to the genomic region upstream of this *Xba*1 site. The *LTL1* knockout DNA fragments detected showed some unexpected size heterogeneity. This appears to be the result of partial transgene deletion, which occurs when a transgene is treated like an IES, that occurred during the crosses that generated these knockout strains (37). The strain that shows the smallest sized *ΔLTL1* band was no longer paromomycin resistant, suggesting that it lost the ability to express the neomycin gene. Additional PCR analyses supported deletion of part of the *NEO3* marker in that strain (data not shown).

To ensure that *ΔLTL1* strains lacked all *LTL1* mRNA expression, we isolated RNA from WT and mutant strains during vegetative growth (V), starvation (S), or during conjugation 3, 6, 9 and 12 h after initiating pairing, and assessed RNA accumulation by reverse transcription (rt)PCR. Consistent with the published microarray data (36), *LTL1* mRNA was first detected in WT cells ∼6 h into conjugation, near the start of post-zygotic development, and continued until at least 12 h (Figure 2B). In contrast, *LTL1* mRNA was not detected in the RNA samples isolated from *ΔLTL1* cells. By using primers specific to the *H*1/*H*P1-like *P*rotein 1 *(HHP1)* coding region, which is expressed at moderate levels at all life-cycle stages (36), we confirmed that transcripts from this control gene were detectable in all samples and thus these mutant strains specifically lacked *LTL1* expression (Figure 2C).

**Figure 3.**
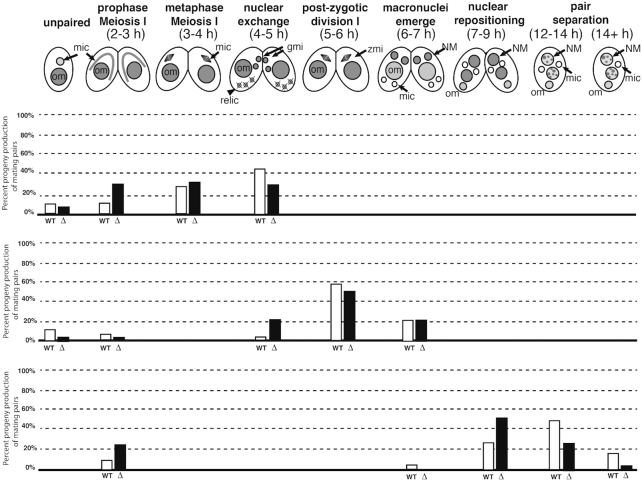
*LTL1* is not required to complete mating and produce progeny. A diagram showing the typical stages and timing of Tetrahymena mating; percentages of paired cells at each time point at the indicated stage: white bars depict data from WT matings, black bars depict *ΔLTL1* data. The progression of each mating was assessed 3 (top), 6 (middle) and 12 h (bottom) after mixing cells. The numbers at each stage was assessed by DAPI-staining DNA visualized by fluorescent microscopy; at least 100 pairs/single cells were counted for each mating at each time point.

To determine whether *LTL1* is essential for development, we crossed WT and *ΔLTL1* strains and compared their abilities to progress through conjugation and produce viable progeny. We collected and fixed WT and *ΔLTL1* mating cells at 3, 6, 9 and 12 h after mixing and then stained their DNA with 4,6-diamidino-2-phenylindole (DAPI) to observe stages of nuclear development. We detected little difference in the progression of WT and mutant cells through defined stages of nuclear development (38) (Figure 3).

To assess the fertility of cells lacking *LTL1, ΔLTL1* strains were crossed to each other or to WT strain CU427 and individual mating pairs were isolated into separate drops of growth media. Mating pairs share cytoplasm, which enables a WT mating cell to rescue many or all defects that might arise from the loss of expression of a disrupted gene in its partner, even in cases when the mutant gene is essential for development. This turned out to be irrelevant as progeny production was similar between mutant × mutant and mutant × WT crosses, ranging between 78% and 91% (Table 1), which is the range we typically observe with WT x WT crosses (20). Thus, Ltl1 is not essential for development.

**Table 1.** Progeny viability of LTL1 knockout strains

Mating strains	# viable (N)	% progeny (N)
ΔLTL1_3-1 x ΔLTL1_5-1	57 (88)	91.2% (52)
ΔLTL1_4-1 x ΔLTL1_5-1	75 (88)	78.7% (59)
CU427 x ΔLTL1_3-1	34 (44)	88.2% (30)
CU427 x ΔLTL1_4-1	40 (44)	92.5% (37)
CU427 x ΔLTL1_5-1	37 (44)	89.2% (33)

**Figure 4.**
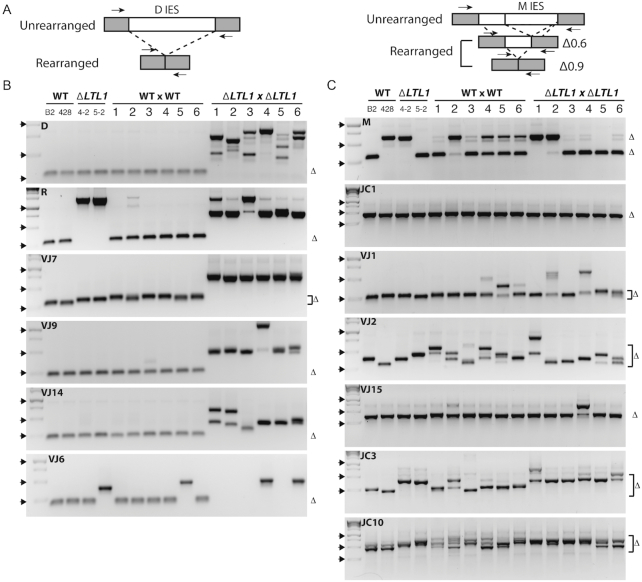
Specific IESs exhibit excision defects in *ΔLTL1* progeny. (**A**) Diagrams depict the rearrangements of loci containing the D and M IESs, examples of IESs whose normal rearrangements produce either a single major form or multiple alternative forms, respectively. Small arrows denote PCR primers flanking each IES used to amplify the loci from genomic DNA. (B, C) PCR amplification of IESs of wild-type, WT progeny, parental *ΔLTL1* cells, and *ΔLTL1* progeny was used to determine the accuracy of IES excision. Delta (Δ) symbols indicate the expected migration of products detected in the WT strains; brackets are used to indicated variable WT boundaries. Lanes B2 and 428 represent amplification of genomic DNA from the two WT strains B2086 and CU428, whose progeny are shown in WT × WT lanes 1–6; *ΔLTL1* represent amplified genomic DNA from the two parental strains 4–2 and 5–2, whose progeny are shown in *ΔLTL1* × *ΔLTL1* lanes 1–6. IES rearrangement was judged to be (**B**) affected or (**C**) unaffected in *ΔLTL1* progeny. Schematic shows the PCR amplified region. The left-most lane contains size standards; solid arrowheads denote the position of migration (250, 500 and 1000 bp fragments). (Note: failure to amplify products in *ΔLTL1* progeny is indicative of aberrant excision resulting in loci that are too large to amplify under the given conditions or loss of sequences complementary to one or both PCR primers).

The observation that *ΔLTL1* cells produced viable progeny indicated that developmentally programmed DNA rearrangements must have occurred because loss of proteins required for DNA elimination results in developmental arrest and cell death (5,29,39–42). This was not unexpected, as strains lacking the related *LIA3* produced viable progeny. To test whether Ltl1 contributes to the accuracy of DNA elimination, we examined the rearrangements of several previously studied IESs, along with the excision events that occur across two selected ∼150 kb regions of the micronuclear genome. About one third of each genomic region is eliminated to produce two ∼100 kb regions of the macronuclear genome. We identified the micronucleus-limited IESs as gaps in the alignments of the micro- and macronuclear sequences, then designed oligonucleotide primers to sequences flanking the ∼15 IESs found within each region to use in PCR to monitor DNA elimination efficiency and accuracy. Of these, we were able to amplify rearrangement products from WT cell genomic DNA for 23 IES-containing loci. For most of these IES-containing loci, PCR amplification produced a single major product, which indicates that DNA elimination in WT cells is reproducibly accurate; however, for some loci, including the well-characterized M IES locus (located in one of the genomic regions analyzed), PCR with flanking primers produced two or more common-sized products, which suggests a normal use of alternative boundaries (Figure 4, Supplementary Figures S2 and S3), and others showed extensive heterogeneity in the size of their rearranged loci in WT cells.

To assess the effect of deleting *LTL1* on IES excision, we crossed WT and *ΔLTL1* strains, isolated DNA from individual progeny lines, and amplified each IES-containing locus. We identified candidate loci which might have boundaries affected by loss of *LTL1* by comparing size ranges of amplified products between *ΔLTL1* parental strains (Supplementary Figure S2, P1-P3) and progeny lines. We examined 34 different loci, including six IESs (IES 1, 2, 3, B, C and D) previously studied in Fass *et al.* (32) and five regulated by Lia3 (20); nine of these 34 were initially classified as possible *LTL1*-regulated candidate loci (Figure S2C and data not shown).

To more clearly assess rearrangement variability, we compared IES excision patterns between individual WT and *ΔLTL1* progeny lines for several candidate and non-candidate loci (Figure 4 and Supplementary Figure S3). Six of these 20 loci showed aberrant rearrangement in *ΔLTL1* progeny that was not apparent in progeny of WT cells. For some of these six, multiple novel excision junctions were produced during development of these *ΔLTL1* progeny (e.g. D IES), evident as multiple PCR products of various sizes, whereas others exhibited a single major PCR product in all progeny indicating that a common set of novel excision junctions resulted from the absence of *LTL1* (e.g. VJ7 IES). This analysis also revealed that some IESs exhibited extensive junction variability in both WT and *ΔLTL1* progeny, which raises the possibility that these loci may not use specific boundary regulators (e.g. VJ2).

We must note that the assignment of the R IES as Ltl1-regulated is complicated by the unexpected presence of unrearranged R IES loci, i.e. retention of this IES in the somatic genomes of two of the *ΔLTL1* parent cells we used in this study. When germline-limited sequences (IESs) are present within a cell's macronucleus, the homologous sequence may be inefficiently eliminated from its progeny (43). Thus, we cannot rule out the possibility that the altered rearrangement patterns observed for this IES is due to epigenetic regulation resulting from intact copies of the R IES in the macronuclei of the *ΔLTL1* parent cells. Because most of *ΔLTL1* progeny show aberrant rather than failed R IES rearrangement and we were able to rescue accurate excison by expression of Ltl1 (Supplementary Figure S8), we remain confident that the boundaries of the R IES are regulated by Ltl1.

To more specifically determine how loss of Ltl1 modifies the rearrangement boundaries of affected IESs, we mapped excision junctions found in the progeny of *ΔLTL1* cells (Supplementary Figure S4; Table 2). For the D IES, we observed multiple sites that were joined to create several novel excision junctions. These new boundary sites were distributed across the IES. In contrast, three IESs that predominantly exhibited a common aberrant rearrangement product in *ΔLTL1* progeny had primarily a single new excision junction. The majority of new boundaries were observed within the region normally eliminated. This may indicate that loss of a boundary regulator generally results in smaller excision events; however, because our PCR primers are located a short distance outside each WT boundary, we would not detect novel junctions positioned in the normally retained region that resulted in removal of sequence corresponding to one or both primers. Furthermore, larger deletions may remove genic or regulatory sequences that affect progeny viability and are therefore not recovered.

**Table 2.** IES boundaries affected in LTL1 knockout strains

IES	IES size	Cell line^a^	Left junction^b^	Right junction^c^
D	1084 bp	P	1	1085
D	680 bp	Δ1L	193	873
D	753 bp	Δ2L	56	809
D	387 bp	Δ2U	262	649
R	1183 bp	P	1	1184
R	627 bp	Δ5	67	694
VJ9	2292 bp	P	1	2293
VJ9	2017 bp	Δ1L	140	2157
VJ7	4753 bp	P	1	4754
VJ7	4142 bp	Δ2U	481	4623

^a^P, IES amplified from Parental line; Δ#, IES amplified from Δ LTL1 progeny, # represents cell line #, letter L and U indicate lower or upper PCR products as migrating on agarose gels.

^b,c^Left and right rearrangement junction numbering correspond to the first and last nucleotide of the IES, respectively.

None of the five IESs previously shown to be regulated by Lia3 (M, 54, 55, 57 and 97) were affected by loss of *LTL1*. To determine whether the subset of IESs regulated by Ltl1 is unique from that regulated by Lia3, we repeated PCR assays for the IESs found to be affected by the absence of *LTL1* using genomic DNA from *ΔLIA3* progeny. We found that none of the IESs regulated by Ltl1 displayed altered excision in the absence of Lia3 (Supplementary Figure S5). Together, these data support our hypothesis that Lia3 and Ltl1 regulate unique subsets of IESs.

### The D IES requires Ltl1 for accurate excision

**Figure 5.**
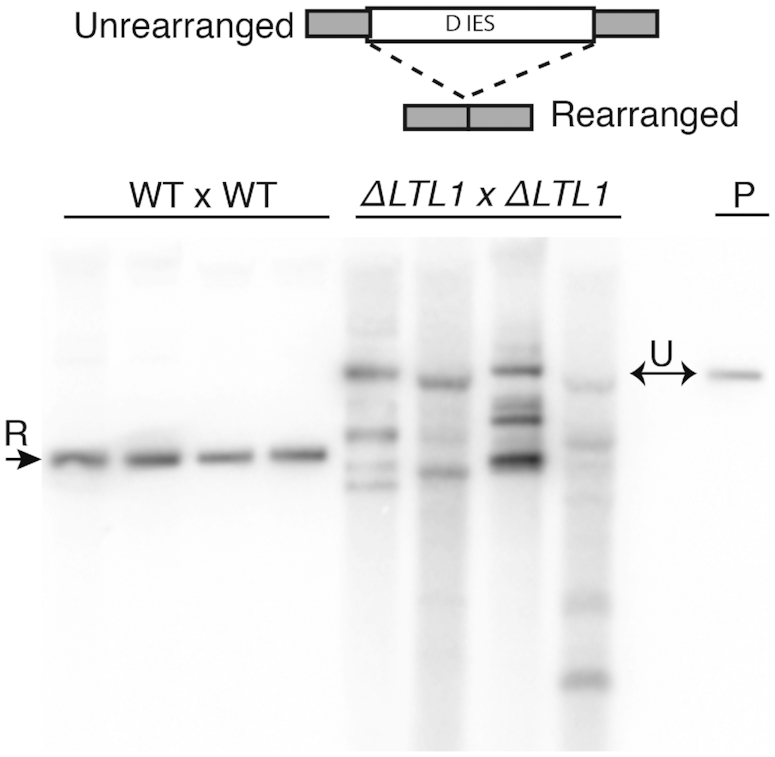
The D IES shows aberrant excision in *ΔLTL1* mutants. Southern blot analysis of DNA isolated from four wild-type (WT) or *ΔLTL1* progeny transformed with a D IES-containing plasmid to assess its rearrangement. A diagram depicting the rearrangement of the D IES is shown at the top. P, digested plasmid DNA used for transformation that shows the migration of the unrearranged (U) D IES. A single rearranged (R) form is visible in WT or multiple aberrant forms in mutant progeny.

Given that DNA rearrangements occur genome-wide, we wanted to ensure that Ltl1 had direct effects on specific IES rearrangement and rule out the possibility that altered rearrangement patterns in *ΔLTL1* cells had resulted from some general perturbation of genome structure affecting excision. To examine IES rearrangement outside the normal genomic context, we cloned the D IES, which exhibited aberrant excision in *ΔLTL1* progeny, onto an rDNA-based replicating vector and introduced the cloned copy into conjugating WT or *ΔLTL1* cells. This plasmid-based rearrangement assay has been long used to study IES excision (14–19). We then isolated DNA from either transformed WT or mutant progeny and examined the structure of the plasmid-borne D IES by Southern blot hybridization. In each WT progeny, excision of the D IES generated the single fragment that was the size expected for accurate excision (Figure 5). In contrast, in each mutant progeny line, multiple fragments were observed, ranging from the size of the unrearranged D IES to sizes consistent with the excision of sequences beyond the normal boundaries (Figure 5). Few, if any fragments were the size expected for the WT excision events, indicating that the D IES was excised with abnormal boundaries in the absence of Ltl1.

**Figure 6.**
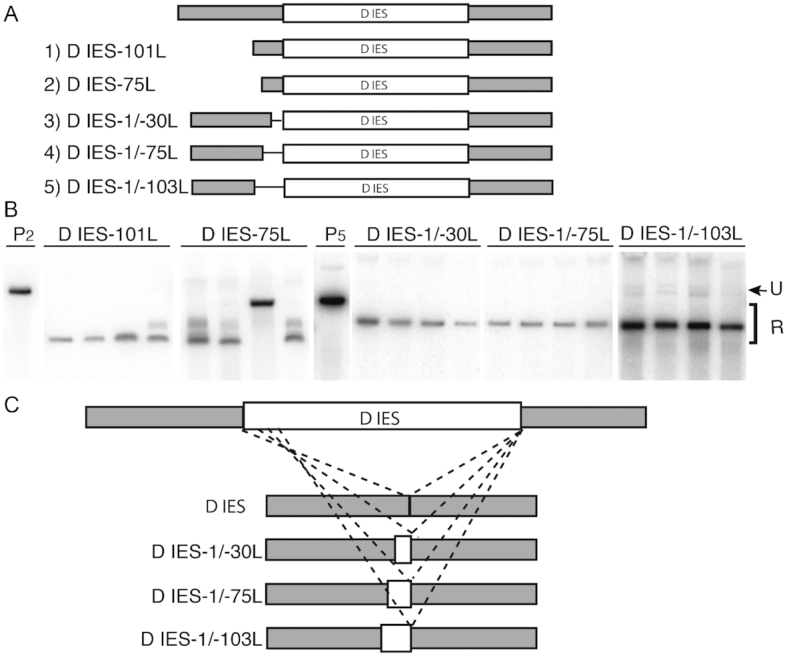
Sequences flanking the D IES position its excision boundaries. (**A**) Deletion mutant constructs used to assess the importance of left-side flanking DNA in the rearrangement of the D IES. The rearrangement of a cloned copy containing several hundred bp of flanking DNA was shown in Figure 5. Constructs 1 and 2 truncate the left flanking DNA, Constructs 3–5 are internal deletions; construct names denote deletion endpoints. (**B**) Southern blot analysis of DNA isolated from progeny transformed with D IES-containing plasmids. P2 and P5, digested plasmid DNA containing constructs 2 and 5 used in the transformation show the migration of the unrearranged (U) D IES; rearranged (R) products migrating below the size of the transformed DNA. (**C**) DNA sequence analysis of internal deletion constructs is illustrated to show that the boundaries shift into the IES roughly the same distance and the sequences removed.

Studies using plasmid-based rearrangement assays have been used to define flanking regulatory sequences, such as the A5G5 motif flanking the M IES, that position the boundaries of excision (14–19). To determine whether the boundaries of the D IES are controlled by flanking regulatory sequences, we monitored the excision of cloned copies with either truncations or small deletions within the left flanking region. Constructs retaining at least 101 bp of the left flank rearranged accurately when transformed into developing *Tetrahymena* cells, whereas those retaining only 75 bp of the left flank exhibited some aberrant excision (Figure 6B). We also examined the rearrangement of D IES constructs for which we deleted 30, 75 or 103 bp immediately outside the left boundary. Each construct produced a single rearranged form, indicating efficient excision of the IES. Even so, when we mapped the excision boundaries, we found that, in each case, the left boundary had shifted to a position approximately equal to the amount of flanking DNA removed (Figure 6C). Together, these results suggest that sequences located 75 bp or more outside the D IES’s left boundary control the cleavage site during excision. Because fully non-overlapping deletions (IESD-75L and IESD-1/-75L) each promoted efficient excision, we could not localize any specific controlling motif. The D IES flanking regulatory sequences may span a relatively long sequence, as observed for the regulatory sequences flanking the R IES (14).

### Ltl1 binds the flanking region of the D IES

To determine whether Ltl1 has DNA binding activity, we purified a maltose binding protein (MBP)-Ltl1 fusion protein, optimized for expression in *E. coli*, to use in electrophoretic mobility shift assays (EMSA). Fusion protein expression was confirmed by western blot analysis using anti-MBP antisera and purification from lysates was assessed by coomassie staining after SDS polyacrylamide gel electrophoresis (Supplementary Figure S6). The full-length MBP-Ltl1 fusion migrated at the expected size of approximately 100 kDa and was the major eluted form. We additionally observed some degradation products. After dialysis, we estimated the concentration of Ltl1 protein (10.5 μM) based on the amount of full-length fusion protein (Supplementary Figure S6).

To investigate the affinity of Ltl1 for DNA, we mixed MBP-Ltl1 with radiolabeled single- or double-stranded (ss or ds)DNA and assessed binding by EMSA. We predicted that Ltl1 would bind to sequences flanking IESs that it regulates, but not to sequences flanking other IESs. We initially tested ss oligonucleotides or ds DNAs from the left flanking region of the D IES (–30 to –88) and the R IES (–40 to –88), which exhibited aberrant or altered excision in *ΔLTL1* progeny, and ds and G-quadruplex (G4) forms of the sequences flanking the M IES, which is regulated by Lia3 not Ltl1 (). We found that Ltl1 consistently bound to ds but not ssDNAs regardless of whether they were sequences flanking an IES regulated by Ltl1 (Supplementary Figure S7 and data not shown).

We did not expect to observe binding to all dsDNA sequences tested. To monitor the affinity of binding, we examined binding across a wide-range of MBP-Ltl1 concentrations to determine the Kd of binding to the ds D IES, R IES, or G4 M IES DNAs (Supplemental Figure S7). We calculated a Kd of approximately 350nM for each substrate. Similar binding assays performed with the addition of 5 μM poly-dGdC as a competitor DNA sequence did not significantly alter the Kd of Ltl1 for these DNAs. Since these substrates were all relatively A+T-rich, it appears that Ltl1 has low-specificity binding affinity for these flanking DNAs.

**Figure 7.**
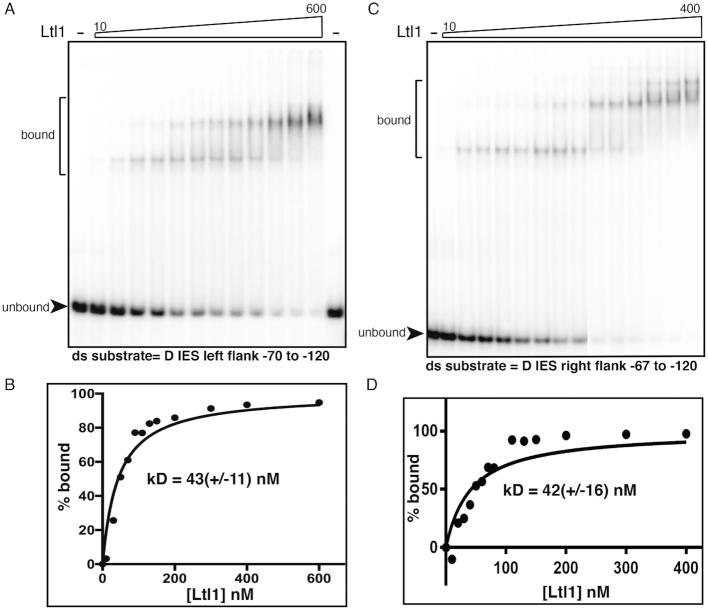
Ltl1 binds dsDNA from the region flanking the D IES. EMSA analysis was performed using increasing amounts of purified MBP-Ltl1 (indicated by the rightward sloping trapezoid) incubated with annealed ^32^P-labeled oligonucleotides corresponding to sequences between (**A**) –120 to –75 of the left flank or (**C**) –120 to –67 of the right flank of the D IES. The position of migration of unbound and bound probe DNA are indicated. (**B, D**) Probe binding was a measure and plotted as the % bound at increasing Ltl1 concentrations.

We initiated these binding studies before we had performed mutagenesis of the D IES that localized important regulatory sequences to the flanking DNA beyond position –75 bp from the left boundary (Figure 6). Our initial binding assays used sequences that corresponded to positions -30 to -88, which, in hindsight explained why this sequence was not preferred over other sequences with similar A+T composition. After testing multiple overlapping sequences, we obtained preferential binding to a dsDNA corresponding to positions –70 to –120 (Figure 7A). We measured a Kd of MBP-Ltl1 binding to this sequence of ∼43 nM, at least 8-fold higher than the low-specificity binding observed to other sequences (Figure 7B). Therefore, both these binding studies and the mutagenesis of the D IES indicate that Ltl1 recognizes an extended region of the flanking sequence of this IES.

Previous studies of the R IES, which Ltl1 appears to regulate, revealed that the corresponding region flanking the right side of this IES can functionally substitute for its left boundary regulatory sequence . Similarly, the essential 5′A_5_G_5_ 3′ motif is located ∼50bp outside each right and left boundary of the Lia3-regulated M IES. These observations led to the hypothesis that the same boundary regulator acts on each side of an IES. To test whether Ltl1 binds to each side of the D IES, we performed binding assays with a radiolabeled dsDNA substrate representing the region from –67 to –120 of the right flanking region (Figure 7C). We observed nearly identical binding affinities (*K*_d_ 42–43 nM) for each substrate (Figure 7B,D). Furthermore, Ltl1 binding caused each substrate to shift from a single bound form to a second form with slower mobility and Ltl1 concentration increased. We conclude that Ltl1 is able to bind sequences on each side of the D IES and regulate its boundaries.

### The conserved regions of Lia3 and Ltl1 are not interchangeable

**Figure 8.**
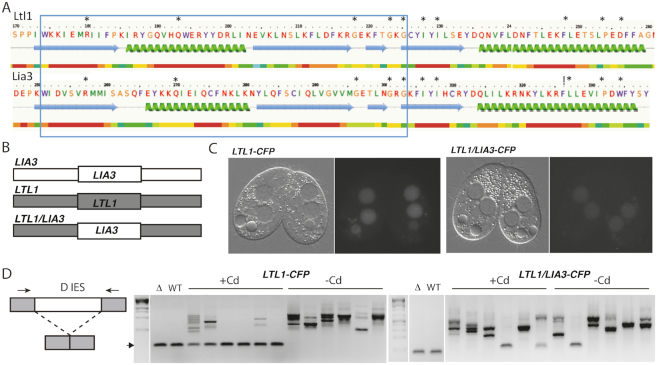
The conserved regions of Ltl1 and Lia3 are not interchangeable. (**A**) Phyre2 structural prediction was used to select the corresponding ∼50 amino acid region (indicated by the large box) to exchange to generate Ltl1/Lia3 chimeric proteins; note very similar predicted structures. Amino acid residues conserved in all four Lia3/Ltl proteins are denoted by asterisks. (**B**) Schematic showing the chimeric constructs that were fused to CFP and transformed into *ΔLTL1* cells. WT and chimeric fusion proteins were expressed from the cadmium (Cd)-inducible *MTT1* promoter. (**C**) Corresponding Differential Interference Constrast images and CFP fluorescence. (**D**) The schematic shows the D IES locus, which was monitored by PCR of genomic DNA isolated from the progeny of transformed cells to the right. Small arrows denote primers used to amplify the locus. Gel electrophoresis of D IES PCR products amplified from genomic DNA isolated from *ΔLTL1* (Δ) or WT strains or the progeny of *ΔLTL1* strains transformed with Ltl1-CFP or the chimeric expression construct as indicated. The arrowhead indicates the expected migration of PCR products corresponding to WT rearrangement products.

We originally identified Ltl1 as a candidate IES boundary regulator because it was similar in both sequence and expression timing to Lia3 (20). To determine whether their similarity in sequence is indicative of functional conservation, we aligned Lia3 and Ltl1 amino acid sequences to define the central conserved region (33) and created a chimeric construct, replacing ∼50 amino acids of Ltl1 with the corresponding region from Lia3 (Figure 8A, B). We then introduced these constructs into *ΔLTL1* strains and assessed whether this chimera could rescue the strains’ IES excision defects. We reasoned that if the Ltl1/Lia3 chimera, with Ltl1 N- and C-termini and the central Lia3 conserved region, could rescue the mutant phenotype, we could conclude that the conserved regions of Lia3 and Ltl1 are functionally equivalent.

To first test whether we could rescue the mutant phenotype, we integrated a CFP-tagged *LTL1* construct, linked to the cadmium (Cd)-inducible *MTT1* promoter, into *ΔLTL1* cells and assessed whether expression of the tagged protein could direct accurate rearrangement of the IESs that display aberrant excision in *ΔLTL1* cells. Upon Cd-induced expression in mating cells, Ltl1-CFP localized within developing macronuclei, where it needs to act, and in degrading parental macronuclei, where it likely accumulated before new macronuclei formed (Figure 8C). Ltl1-CFP expression (+Cd) was sufficient to restore accurate IES rearrangement of the D IES (Figure 8D) and other Ltl1-regulated IESs (Supplementary Figure S8) whereas multiple aberrant deletion products were detected in *ΔLTL1* progeny without induction (–Cd). The rescue of defective IES excision by Ltl1-CFP expression provides further evidence that Ltl1 is essential to guide accurate DNA elimination at these loci.

We next inserted a chimeric *LTL1/LIA3*-CFP construct into *ΔLTL1* mutant cells and induced expression of the CFP-tagged protein. The chimeric protein localized to developing macronuclei much like Ltl1-CFP (Figure 8C). Nevertheless expression of Ltl1/Lia3-CFP was insufficient to rescue accurate IES excision. In all cases, induced expression of the chimeric-CFP protein resulted in progeny with similar degrees of IES excision heterogeneity as was observed in the progeny of uninduced *ΔLTL1* mutants (Figure 8D; Supplementary Figure S8). Despite the sequence and structural similarity of the corresponding conserved regions of these two boundary regulators, amino acids from Lia3 do not effectively substitute with the corresponding region of Ltl1.

## DISCUSSION

Through this study, we showed that *LTL1* encodes a boundary regulatory protein that directs accurate excision of a subset of IESs. Thus, the amino acid similarity between Ltl1 and Lia3 (Figure 1; (20)) is indicative of their analogous functional roles during formation of the somatic genome. Even so, we found that Ltl1 has very different DNA binding properties than Lia3, which binds specifically to G quadruplex DNA. Ltl1 has a general affinity (Kd = ∼350 nM) for dsDNA and shows specific binding (*K*_d_ = ∼43 nM) for a 50 bp sequence from the region flanking the D IES; Lia3 does not bind dsDNA (20). The regions of similarity between these two proteins, which span one quarter of each protein, are sufficiently different such that a chimeric Ltl1/Lia3 protein could not substitute for the wild type protein (Figure 8). Therefore, despite their similarities, these proteins effectively bind different DNA substrates.

We examined how rearrangement was affected in the progeny of *ΔLTL1* cells for more than two-dozen IESs and found that ∼18% of these IESs showed irregular excision patterns. For some IESs, such as the D IES, loss of *LTL1* resulted in multiple novel rearrangement junctions, but for others a predominant non-wild-type deletion event occurred. IESs that showed multiple deleted forms appear to have lost of all ability to position boundaries when Ltl1 is not present. For the others, it would appear that the genome retains some ability to direct the accuracy of excision upon loss of the primary control protein. It is possible that these altered junctions reflect a preferential pattern by which scan RNAs target chromatin modifications to these IESs. The excisase Tpb2 interacts with methylated histone H3, and this interaction may be sufficient to select preferred alternate boundaries (44). Alternatively, these IESs may possess cryptic binding sites for other boundary regulators that get recruited upon loss of Ltl1. This second possibility may reveal the evolutionary history of an IES. IESs are prone to insertion/deletions (45,46). As IESs expand or contract, the major boundaries may shift even though sequences recognizable by different boundary regulators remain within these loci.

Most of the IESs we examined were located in two regions of the genome, and they represent a small fraction of the ∼12 000 IES (4); therefore, we do not have a good basis to estimate the actual number of the thousands of IESs that might be controlled by Ltl1. The IESs affected by loss of Ltl1 were clearly distinct from those controlled by Lia3, which is consistent with our hypothesis that IESs can be grouped into families based on the specific regulatory protein that they use to position their boundaries. We speculate that, by requiring the use of the same boundary-regulating protein on both sides of an IES and different boundary-regulating proteins for adjacent IESs, *Tetrahymena* effectively prevents deleterious deletions that would occur if the distal ends of two neighboring IES-containing loci were to be joined.

Structural prediction using Phyre2 revealed that Ltl1 has an amino-terminal helix-turn-helix motif that is similar to the bipartite DNA binding domain of the Tc3 transposase and other DNA binding proteins; Lia3 lacks this motif. The bipartite DNA binding domain of the Tc3 transposase acts as a dimer, and loops the ends of the transposable element together (47). This action is consistent with the use of the same boundary regulator on each side of an IES. The EMSA pattern of DNA/Ltl1 binding exhibits a second upward shift at higher protein concentrations that could be indicative of dimerization after binding (Figure 7). The helix-turn-helix motif of this structure can mediate both specific and non-specific interactions with the major groove of the DNA (48). This motif may account for some or all of the overall affinity for DNA, but as it comprises only one-quarter of the of the Ltl1 protein, other parts of the protein may contribute to Ltl1 binding specificity. If multiple regions of the protein are critical for binding, it may explain why the Ltl1/Lia3 chimera was not functional.

A transposon origin for IESs has long been suspected (49), and the similarity between Ltl1 and the DNA-binding motif of Tc3 transposases may be further evidence that IESs are the remnants of transposons. Transposases act on the ends of their elements and deposit their DNA binding sites at transposon termini upon insertion into a host's genome. The IESs controlled by any specific boundary regulator may have originated from insertions of a common transposon. By domesticating the transposon's end-binding proteins to control IES excision, the rest of the transposon sequence can fade, no longer easily recognized as a transposon. These domesticated boundary regulators no longer need to retain transposase activity as its role is served by Tpb2 (and Tpb1 for a unique subset of IESs) (50–52).

The specificity of Ltl1 for a long A+T rich sequence in the flank of the D IES is quite different from the G quadruplex binding of the related protein Lia3. Despite their different binding affinities, both these proteins determine the accuracy of IES excision. We envision two ways by which these proteins may direct the excision accuracy. The first is by directly recruiting Tpb2 to the ends of an IES. Alternatively, they may act by limiting the spread of the RNA-directed chromatin modifications. This second possibility is intriguing as it would suggest that these regulators create individual chromatin domains, which mimics the way that a chromatin boundary regulator acts in genomes that do not undergo programmed DNA elimination. Further investigation into the mechanism(s) by which these proteins coordinate accurate cleavage on each side of an IES will likely elucidate the role of chromosome architecture in these events and provide new insights into the organization of distinct chromatin domains.

## Supplementary Material

gkz504_Supplemental_File

## Notes

Present address: Vita N. Jaspan, New York University School of Medicine, New York, NY 10016, USA.
