# Supplementary material for: Boundaries of eliminated heterochromatin of Tetrahymena are positioned by the DNA-binding protein Ltl1
Source: Nucleic Acids Res. 2019 Jun 13;47(14):7348–62. doi: 10.1093/nar/gkz504 (PMC6698652; doi:10.1093/nar/gkz504)
Supplement: gkz504_Supplemental_File [file gkz504_supplemental_file.pdf]

SUPPLEMENTARY TABLES AND FIGURES

Supplementary Table 1: Oligonucleotide Primers Used for Generating LTL1 Constructs

Name	Purpose	Sequence (5'→3')
**Creating LTL1 KO Construct**		
499_KOup_FW	Amplify upstream flank of LTL1	AGGTACCAAAATCAGACGATTGG
499_KOup_RV	Amplify upstream flank of LTL1	GGCGCGCCTAGGCGTACGTCGACAA
499_KOds_FW	Amplify downstream flank of LTL1	GTCGACGTACGCCTAGGCGCGCCTCATTTTA CAAAACAGCCAACA
499_KOds_RV	Amplify downstream flank of LTL1	AGGTACCTTTTTGCTTGGTTTATGTG
**Creating LTL1/Lia3 Chimeras**		
Lia3_3'StopBm	amplify whole Lia3 coding region	GGATCCTCAGAATAAACTGTTCAGAGGT
Lia3_-6gtw	Amplify the whole Lia3 coding region	CACCACTCAAATGAAATCTCATTCAAAGAAA TACC
499370_3'_bam	amplify whole Ltl1 coding region	GGATCCATTTAAAACGTCTTCTTATTTTGC
00499370_FW	Amplify whole LTL1 coding region	CACCATAACCATGAATTTAACTTCTTAGCCT ATAAACATAGA
5'lia3/499_R	connect Lia3 flanks/Ltl1 conserved region	GATCATATCTACAATGGATATAAATGAAT
5'lia3/499_L	connect Lia 3 flank/Ltl1 conserved region	GGTTAGTAATCTGATGAGCCAAAAATGGA
5'499/Lia3_L	connect Ltl1 flank/Lia3 conserved region	AGACCCTTAATCGCCTCCCATTTGGATT
5'499/Lia3_R	connect Ltl1 flanks/Lia3 conserved region	TCATATTCAGACAAAATGTAAATATAGCA
5'499L	amplify Ltl1 conserved region	CAAGAACCAAGTCCGATAGACC
5'499R	amplify Ltl1 conserved region	TCTTTCCCACTAATGAACTTAGCC
5'Lia3L	amplify Lia3 conserved region	CACAATTCGAAGAAGTCAAAGG
5'Lia3R	amplify Lia3 conserved region	GCTTGCTGAAATCATCATTCTT
5'lia3/499 L rev	amplify Lia3 flanks/pENTR plasmid	GGAAAATAATTCTCATTTCAATCTTTTTCC
5'Lia3/499 R rev	amplify Lia 3 flanks/pENTR plasmid	GAGAAAAATTTACTGGTAAAGGCAAATT
5'499/Lia3 L rev	amplify Ltl1 flanks/pENTR plasmid	TCATTCTTACACTGACATCAATCCAAATG
4'499/Lia3 R rev	amplify Ltl1 flanks/pENTR plasmid	GTGAAACACTTAATGGAAGAGGATGCTA

Supplementary Table 2 Oligonucleotide Primers Used to Confirm LTL1 Knockout Cell Lines

Name	Purpose	Sequence (5'→3')
p452-3351	Screening for LTL1 mutants (upstream)	TCGCCTTCTTGACGAGTTCT
499_KOds_RV	Screening for LTL1 mutants (downstream)	AGGTACCTTTTTGCTTGGTTTATGTG
499370_WT_fw	upstream primer for screening for WT LTL1	ATCGCCTCCCATTTGGTAAT
499370_WT_rv	downstream primer for screening for WT LTL1	ATTAAGGCAATTCCCAGCAA
499370_up	upstream primer to confirm correct location of KO construct	AGGTAAAGATATTTCGCATTTTT
499370_ds	Downstream primer to confirm correct location of KO construct	TGATTCGCTGTTCACATTCC

Supplementary Table 3: Oligonucleotide Primers Used to Assay IES Excision

Name	Purpose	Sequence (5'→3')
IES11_MDSL-42	Upstream primer for D IES	GGCCACAATATACTAAGGCAATTT
IES11_MDSR-34	Downstream primer for D IES	GGCCACCTTGATACCAGTTT
R5'(168-sense)-2	Upstream primer for R IES	AATTATTCTTTATAATCTGACTC
R3'(1453-anti)-2	Downstream primer for R IES	AAGATAGTTCTAGAATAAGAC
M1194	Upstream primer to detect M IES excision	GTGGGGAGGGAGAAGGATTCAAC
M002	Downstream primer to detect M IES excision	ACCTTAAACAAATGCCATATTGAG
IES_VNJ1_up	Upstream primer for VJ1 IES	GCAAGTTACTTGTAAAGTATTTTTAAT
IES_VNJ1_dwn	Downstream primer for VJ1 IES	AACCTAAAATATAGATAGGTAGGTAGA
IES_VNJ2_up	Upstream primer for VJ2 IES	CACCAAGCATTCCATTCAT
IES_VNJ2_dwn	Downstream primer for VJ2 IES	TCATAATCAAAAAGATTAGTTGTCT
IES_VNJ4_up	Upstream primer for VJ4 IES	TTTTTGTTTCTGTGTTTACTAAAAT
IES_VNJ4_dwn	Downstream primer for VJ4 IES	CCAACCAACCATTAATGACA
IES_VNJ5_up	Upstream primer for VJ5 IES	TTTTCAATTATCAACTCAGAAAAA
IES_VNJ5_dwn	Downstream primer for VJ5 IES	TGTAATTCAGCCTAGGGTTATAAAAT
IES_VNJ6_up	Upstream primer for VJ6 IES	ATGGTTTAATAATCTTTTAAATAATACA
IES_VNJ6_dwn	Downstream primer for VJ6 IES	CATTGGATATAACTTCCGTAAATC
IES_VNJ7_up	Upstream primer for VJ7 IES	CAATCACCAATCACTATGCTTTT
IES_VNJ7_dwn	Downstream primer for VJ7 IES	TCAGGAGATTCACACATTATTGA
IES_VNJ8_up	Upstream primer for VJ8 IES	AAAAATTACAAATAAGGAGTCAAAA
IES_VNJ8_dwn	Downstream primer for VJ8 IES	AATAAACTTTGTTTACCCAAAAA
IES_VNJ9_up	Upstream primer for VJ9 IES	ACATCTCCGAATTGGTTGA
IES_VNJ9_dwn	Downstream primer for VJ9 IES	AACATTCCTTATTAATTAACTCTCC
IES_VNJ10_up	Upstream primer for VJ10 IES	CATTTTTCATCTTTAATACCAATTTTCA
IES_VNJ10_dwn	Downstream primer for VJ10 IES	TCCTGCAAAATTATTCCTCATT
IES_VNJ12_up	Upstream primer for VJ12 IES	AAACAGTGTAAAACCCAAAAAGC
IES_VNJ12_dwn	Downstream primer for VJ12 IES	TGCTTAAACCATTTTGTTATTTCG
IES_VNJ13_up	Upstream primer for VJ13 IES	CGTTGATTTATTTTGGTATCTGA
IES_VNJ13_dwn	Downstream primer for VJ13 IES	TTTTAGGGCGAATCACCTTT
IES_VNJ14_up	Upstream primer for VJ14 IES	TGGTGCTCAAATACAATTCTCAA
IES_VNJ14_dwn	Downstream primer for VJ14 IES	TCGTTTTCGCTCATTTTTCT
IES_VNJ15_up	Upstream primer for VJ15 IES	CAAAAATCTAGTGTTGATTGAATACC
IES_VNJ15_dwn	Downstream primer for VJ15 IES	TTCAAAGCATTGCTCATTCTG
IES_JC1_up2	Upstream primer for JC1 IES	TTGCTAGGATGAAATGTAATTAGGC
IES_JC1_ds	Downstream primer for JC1 IES	CCTTGAATGTAACCTTAAGCTCAGT
IES_JC2_up	Upstream primer for JC2 IES	AACCTTACAAGCATTTTGTGCAT
IES_JC2_ds	Downstream primer for JC2 IES	AGATGCCCTGATGGATGTTC
IES_JC3_up	Upstream primer for JC3 IES	TCCTTCCTTACTTACTTACCTTGGA
IES_JC3_ds	Downstream primer for JC3 IES	TCATTGAATAATCAAACTTTCGTTTC
IES_JC4_up	Upstream primer for JC4 IES	TTGAAAGCTTGAATTCCTTTTTCT
IES_JC4_ds	Downstream primer for JC4 IES	AATTTGTTTAATTCCTGGATTTGC
IES_JC5_up	Upstream primer for JC5 IES	TGATTATTTGGATAGGACACTGAAA

IES_JC5_ds	Downstream primer for JC5 IES	ACCAAAAATGCAAATAAAATTGGT
IES_JC6_up	Upstream primer for JC6 IES	TAATTTGGCTAAATGTCAAAAGCA
IES_JC6_ds	Downstream primer for JC6 IES	TAAGGCCACCTTGATACCAGTTTA
IES_JC7_up	Upstream primer for JC7 IES	GCGTAATATAGTCAATTCCCTACTCA
IES_JC7_ds	Downstream primer for JC7 IES	CATCAAAATAAAATAGAACTGATTTGAAAC
IES_JC8_up	Upstream primer for JC8 IES	ATTAGTTTTGTTGTCGGCCATACT
IES_JC8_ds	Downstream primer for JC8 IES	ATTTGAGTAAGCATAAACCCGAAT
IES_JC9_up	Upstream primer for JC9 IES	TGTAAAATGATGCAGGGATTAAAA
IES_JC9_ds	Downstream primer for JC9 IES	TGCCATATATTTGTTGATCCAGTT
IES_JC10_up	Upstream primer for JC10 IES	TTATTTGAAGGGTCTTCAGGGATA
IES_JC10_ds	Downstream primer for JC10 IES	TTTTGCAATAACTGCTGATCAAAT
IES_JC11_up	Upstream primer for JC11 IES	ATGAAAGAGCCTTCCATTTTTAAT
IES_JC11_ds	Downstream primer for JC11 IES	CGACTCTGATTTCCAAGTTCTTTT
IES_JC12_up	Upstream primer for JC12 IES	TGTAAAATGATGCAGGGATTAAAA
IES_JC12_ds	Downstream primer for JC12 IES	TTATCTCTGTTGCTGAAGTTGGAG

Supplementary Table 4: Oligonucleotide Primers Used for Generating LTL1 Expression construct

Name	Purpose	Sequence (5'→3')
LTL1s_r	Upstream primer for amplification of LTL1s	CACCGAATTCGGATCCGAGA
LTL1s_L	Downstream primer for amplification of LTL1s	GCCAGTGCCAAGCTTAGTTCA
LTLs_scn_R	Upstream primer for screening for LTL1s	ACCAGGAAATGACCGTCAAC
LTLs_scn_L	Downstream primer for screening for LTL1s	ATTTTTCGCCACGTTTGAAG

Supplementary Table 5: Oligonucleotide Probes Used in EMSA Experiments

Name	Purpose	Sequence (5'→3')
IESD_EMSA_L-70/120	D IES EMSA substrate left flanking region -70 to -120	AAAATTTATATATATTTGAATCCTAAGAT<br>TCTGTCTTATTTGATTAATTAA
IESD_EMSA_L-70/120	Reverse complement to D IES EMSA substrate left flanking region -70 to -120	TTAATTAATCAAATAAGACAGAATCTTAG<br>GATTCAAATATATATAAATTTT
IESD_EMSA_R-67/120	D IES EMSA substrate right flanking region -67 to -120	TTTGAATCCTAAGATTCTACTTTATTTAG<br>CATTTTTTATATTAAGTAATTTA
IESD_EMSA_ R-67/120	Reverse complement to D IES EMSA substrate right flanking region -67 to -120	TAAATTACTTAATATAAAAAATGCTAAAT<br>AAAGTAGAATCTTAGGATTCAAA
R_IES_EMSA_probe2	R IES EMSA substrate left flank -40 to -88	GAAATTAATATAATTATAATTTTAAACAGTGT<br>AAAACCCAAAAAGCTA
R_ES_EMSA_probe2rc	R IES EMSA substrate left flank -40 to -88 (reverse complement)	TAGCTTTTTGGGTTTTACACTGTTTAAAATTA<br>TAATTATATTAATTTC
IESD_EMSA_probe1	D IES EMSA substrate left flank -30 to -88	TTATTTGATTAATTAATTTTATTTACGGTAAA<br>GGTTTATATATTAAGGCCACAATATAC
IESD_EMSA_probe1rc	D IES EMSA substrate left flank -30 to -88 (reverse complement)	GTATATTGTGGCCTTAATATATAAACCTTTAC<br>CGTAAATAAAATTAATTAATCAAATAA
M1_A5G5_FW	M IES EMSA substrate left flank -34 to -63	TTTATTAATCAAAAAGGGGGTAAATAATAA
M1_A5G5_RV	M IES EMSA to make ds probe	TTATTATTTACCCCCTTTTTGATTAATAAA

SUPPLEMENTARY FIGURE LEGENDS

Supplementary Figure S1: Confirmation of Ltl1 Knockout Strains. PCR amplification of *Tetrahymena* genomic DNA isolated from *ΔLTL1* and wild type (WT) strains using primers unique to (A) LTL1 or to (B) the *ΔLTL1* allele. Location of primers is shown in the schematics above each gel image. (A) PCR of WT strain DNA (CU428 and B2086) amplifies the expected 339bp product while and PCR of *ΔLTL1* strain DNA produces the predicted 1380bp product.

Supplementary Figure S2: Some IESs exhibit excision defects in *ΔLTL1* progeny. A) Diagrams depict the rearrangements of loci containing the D and M IESs, examples of IESs whose normal rearrangements produce either a single major form or multiple alternative forms, respectively. Small arrows denote PCR primers flanking each IES used to amplify the loci from genomic DNA. B, C) PCR amplification of IESs in parental *ΔLTL1* cells and their progeny was used to determine the accuracy of IES excision. IES designation is given in left corner of each gel image. IESs were judged to: B) be largely unaffected, or C) exhibit possible altered rearrangement need for additional study. Delta (Δ) symbols indicate the expected migration of products detected in the parents and wild-type strains (from analysis not shown). Lanes P1-P3, amplification of genomic DNA from the three *ΔLTL1* parent strains, lanes 1-5 amplification of genomic DNA from five *ΔLTL1* progeny. Solid arrowheads, the position of migration of size standards (bottom to top-250, 500,and 750bp).

Supplementary Figure S3: IESs with excision defects in *ΔLIA3* progeny are unaffected in *ΔLTL1*progeny. A, B) PCR amplification of IESs using genomic DNA from WT strains, WT progeny, parental *ΔLTL1* cells, and *ΔLTL1* progeny was used to determine the accuracy of IES excision. IESs examined include those (54, 55, 57, and 97) previously shown to have altered rearrangement patterns in *ΔLIA3* progeny. Lanes B2 and 428 represent amplification of genomic DNA from the two WT strains B2086 and CU428, whose progeny are shown in WT x WT lanes; *ΔLTL1* represent amplified genomic DNA from the two parental strains 4-2 and 5-2, whose progeny are shown in *ΔLTL1* x *ΔLTL1* lanes. Solid arrowheads, the position of migration of size standards (bottom to top-250, 500,and 1000bp). IES designation is given in left corner of each gel image.

Supplementary Figure S4: Altered boundaries of IES excision in *ΔLTL1* progeny. The schematics represent the IESs examined and labeled on the right. Selected rearrangement products were gel purified and sequenced. Boundaries are shown as colored arrows on the schematics next to the gel, as compared to WT boundaries, in green. The colors of the arrows on the schematics denote junctions of the corresponding sequenced product indicated by the same colored arrow on the gel image. Not all products analysed are not shown on the gel images shown. P1-P3, products amplifed using DNA from parental *ΔLTL1* strains; 1-5 , products amplifed using DNA from *ΔLTL1* progeny lines.

Supplementary Figure S5: IESs with excision defects in *ΔLTL1* progeny are unaffected in *ΔLIA3* progeny. A) Diagram depict the rearrangements of a generic IES locus whose normal rearrangements produce a single major form. Small arrows denote PCR primers flanking each IES used to amplify the loci from genomic DNA. B, C) PCR amplification of IESs in eight *ΔLIA3* progeny was used to determine the accuracy of IES excision: B) IESs largely unaffected

in *ΔLTL1* progeny, or C) IESs that exhibit altered rearrangement in *ΔLTL1* progeny. Delta (Δ) symbols indicate the expected migration of products detected in wild-type strains (from analysis not shown). Solid arrowheads, the position of migration of size standards (bottom to top-250, 500,and 750bp). IES designation is given in left corner of each gel image.

Supplementary Figure S6: Estimation of the concentration of Purified MBP-Ltl1. Purified proteins were visualized by coomassie-staining after electrophoresis of purified MBP-Ltl1 and known amounts (given above each lane) of Bovine Serum Albumin (BSA) on a SDS polyacrylamide. The amount of isolated full-length MBP-Ltl1 was estimated based comparison to the BSA standard. The sizes of pre-stained protein markers are indicated on the left in kDa.

Supplementary Figure S7: Ltl1 binds to double-stranded DNA. EMSA using Ltl1 and radiolabeled substrates corresponding to regions flanking IESs. A) The diagram shows the position of binding substrates corresponding to sequences flanking the left side of the R, D, or M IES. B) EMSA using single-stranded R IES (ssR), double-stranded R IES (dsR), single-stranded D IES (ssD), double-stranded D IES (dsD), double-stranded M IES (dsM), and G-quadruplex (G4 M) for the M IES mixed with increasing amounts of MBP-Ltl1 (0, 1100, or 2100 nM) as indicated above each set of wells. The asterisk indicates that the majority of probe used in these reactions had formed a G-quadruplex. Brackets denote the migration of unbound and bound substrates. C) and D) EMSA shows binding upon titration with increasing amounts (0, 25, 50, 75, 100 150, 200, 50, 300, 400, 500, 700, 900, 1100, 1500, 2100nM) of MBP-Ltl1, which was used to determine Kd of Ltl1 binding to C) dsR(-88/40) and d) dsD(-88/30) IES left flanking DNA substrates.

Supplementary Figure S8. Expression of Ltl1-CFP rescues IES excision defects in *ΔLTL1* progeny. A) The diagram indicates the intact LTL1 coding region was fused to CFP and transformed into *ΔLTL1* cells. After expression from the cadmium (Cd)-inducible *MTT1* promoter the ability of the tagged protein to rescue excision defects was assessed by PCR. B) Diagram shows an general IES locus, which was monitored by PCR of genomic DNA isolated from the progeny of transformed cells with (+Cd) or without (-Cd) induced expression. Small arrows denote primers used to amplify the locus. C) Gel electrophoresis of PCR products that detect six IESs amplified from genomic DNA isolated from: a *ΔLTL1* (Δ) strain, a WT strain, or the progeny of *ΔLTL1* strains transformed with Ltl1-CFP. The arrowhead in B) and C) indicates the expected migration of PCR products corresponding to WT rearrangement products.

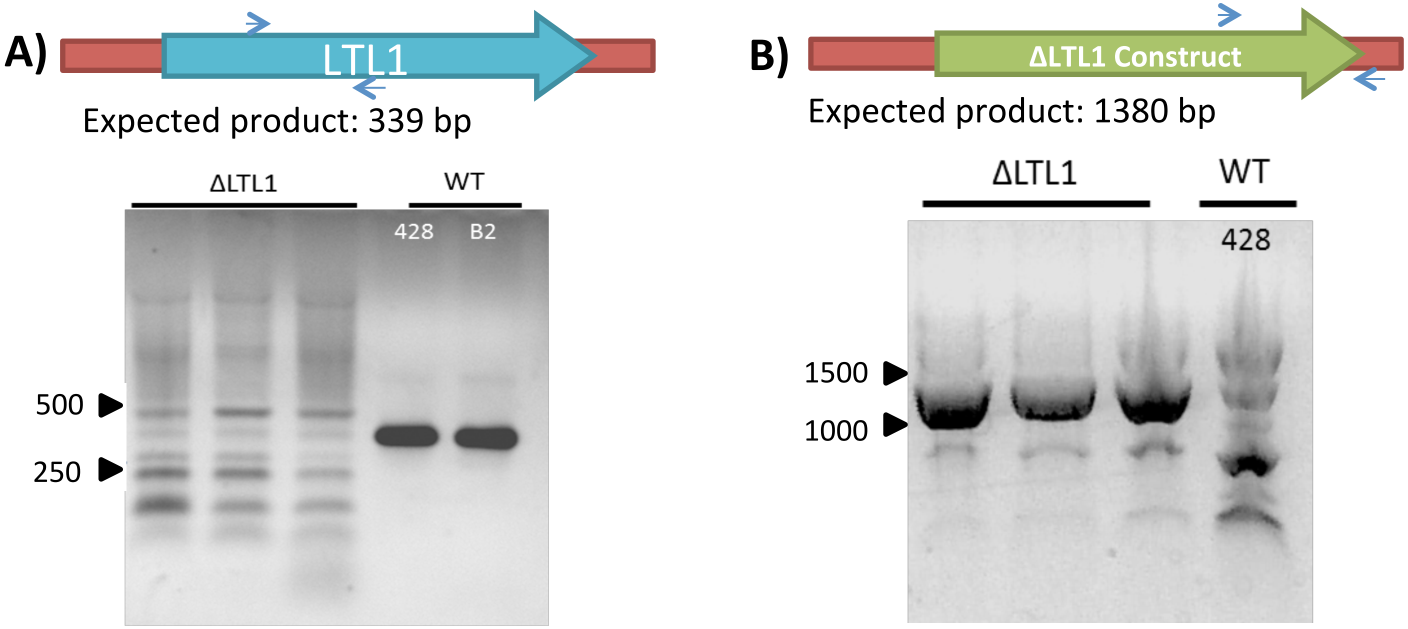

Supplementary Figure S1: *Confirmation of Ltl1 Knockout Strains*. PCR amplification of *Tetrahymena* genomic DNA isolated from *ΔLTL1* and wild type(WT) strains using primers unique to (A) *LTL1* or to (B) the *ΔLTL1* allele. Location of primers is shown in the schematics above each gel image. (A) PCR of WT strain DNA (CU<u>428</u> and <u>B2</u>086) amplifies the expected 339bp product while and PCR of *ΔLTL1* strain DNA produces the predicted 1380bp product.

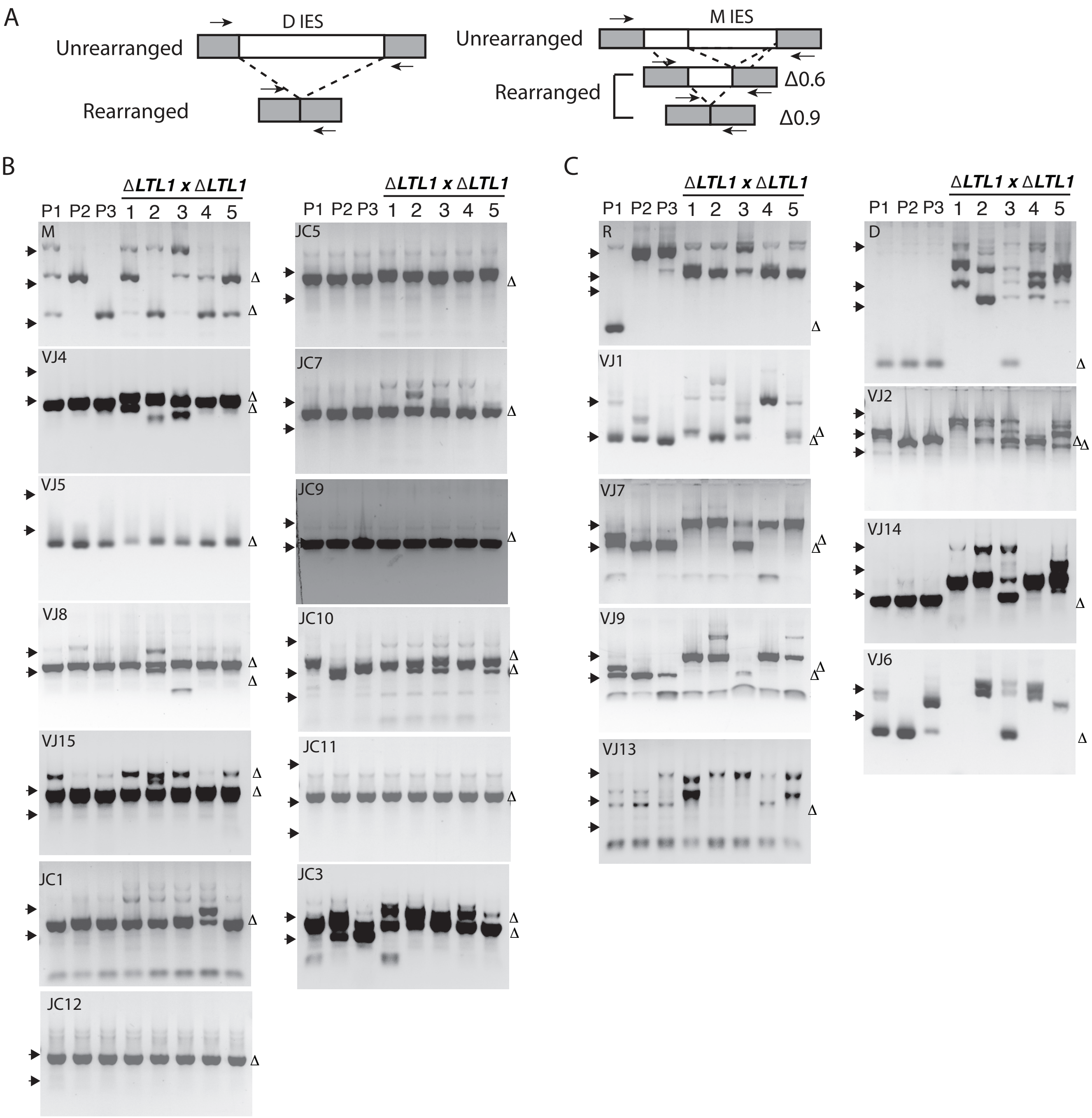

Supplementary Figure S2: Some IESs exhibit excision defects in *ΔLTL1* progeny. A) Diagrams depict the rearrangements of loci containing the D and M IESs, examples of IESs whose normal rearrangements produce either a single major form or multiple alternative forms, respectively. Small arrows denote PCR primers flanking each IES used to amplify the loci from genomic DNA. B, C) PCR amplification of IESs in parental *ΔLTL1* cells and their progeny was used to determine the accuracy of IES excision. IES designation is given in left corner of each gel image. IESs were judged to: B) be largely unaffected, or C) exhibit possible altered rearrangement need for additional study. Delta (Δ) symbols indicate the expected migration of products detected in the parents and wild-type strains (from analysis not shown). Lanes P1-P3, amplification of genomic DNA from the three *ΔLTL1* parent strains, lanes 1-5 amplification of genomic DNA from five *ΔLTL1* progeny. Solid arrowheads, the position of migration of size standards (bottom to top-250, 500,and 750bp).

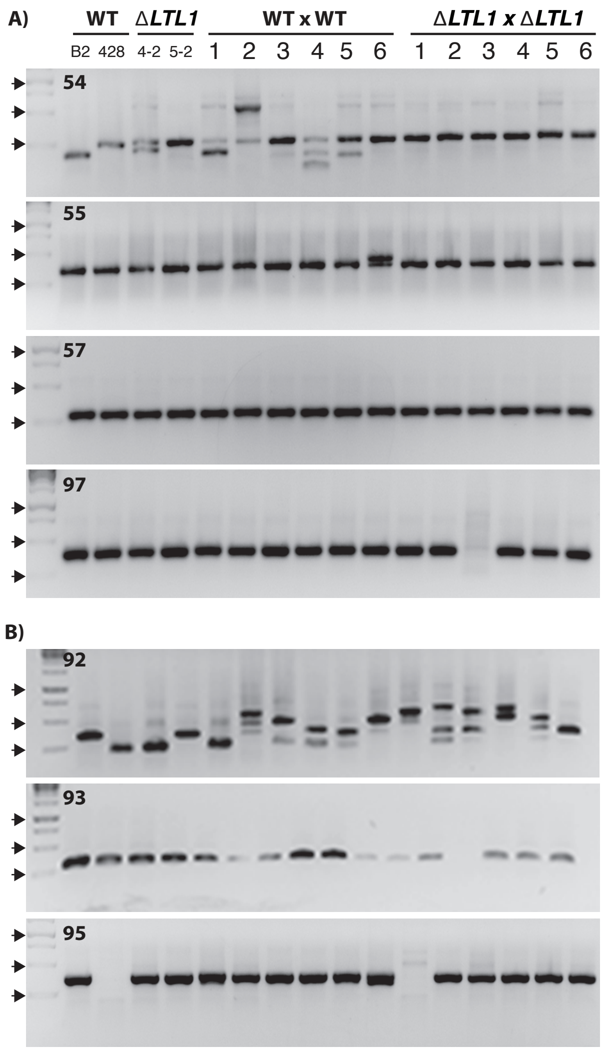

Supplementary Figure S3: IESs with excision defects in *ΔLIA3* progeny are unaffected in *ΔLTL1* progeny. A, B) PCR amplification of IESs using genomic DNA from WT strains, WT progeny, parental *ΔLTL1* cells, and *ΔLTL1* progeny was used to determine the accuracy of IES excision. IESs examined include those (54, 55, 57, and 97) shown in (A) previously shown to have altered rearrangement patterns in *ΔLIA3* progeny; B) IES that are unaffected by loss of Lia3. Lanes B2 and 428 represent amplification of genomic DNA from the two WT strains B2086 and CU428, whose progeny are shown in WT x WT lanes; *Δ LTL1* represent amplified genomic DNA from the two parental strains 4-2 and 5-2, whose progeny are shown in *ΔLTL1 x ΔLTL1* lanes. Solid arrowheads, the position of migration of size standards (bottom to top-250, 500,and 1000bp). IES designation is given in left corner of each gel image.

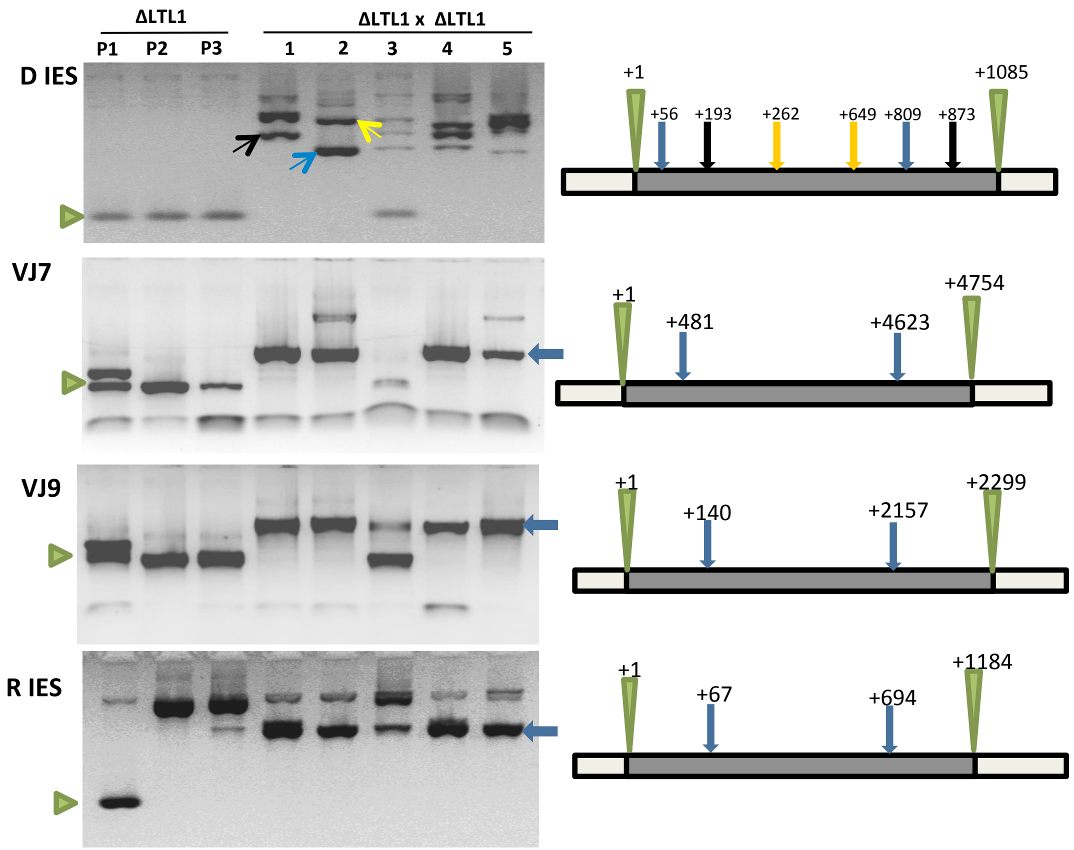

Supplemental Figure S4: **Altered boundaries of IES excision in *ΔLTL1* progeny.** The schematics represent the IESs examined and labeled on the right. Selected rearrangement products were gel purified and sequenced. Boundaries are shown as colored arrows on the schematics next to the gel, as compared to WT boundaries, in green. The colors of the arrows on the schematics denote junctions of the corresponding sequenced product indicated by the same colored arrow on the gel image. Not all products analysed are shown on these gel images. P1-P3, products amplifed using DNA from parental ΔLTL1 strains; 1-5, products amplifed using DNA from ΔLTL1 progeny lines.

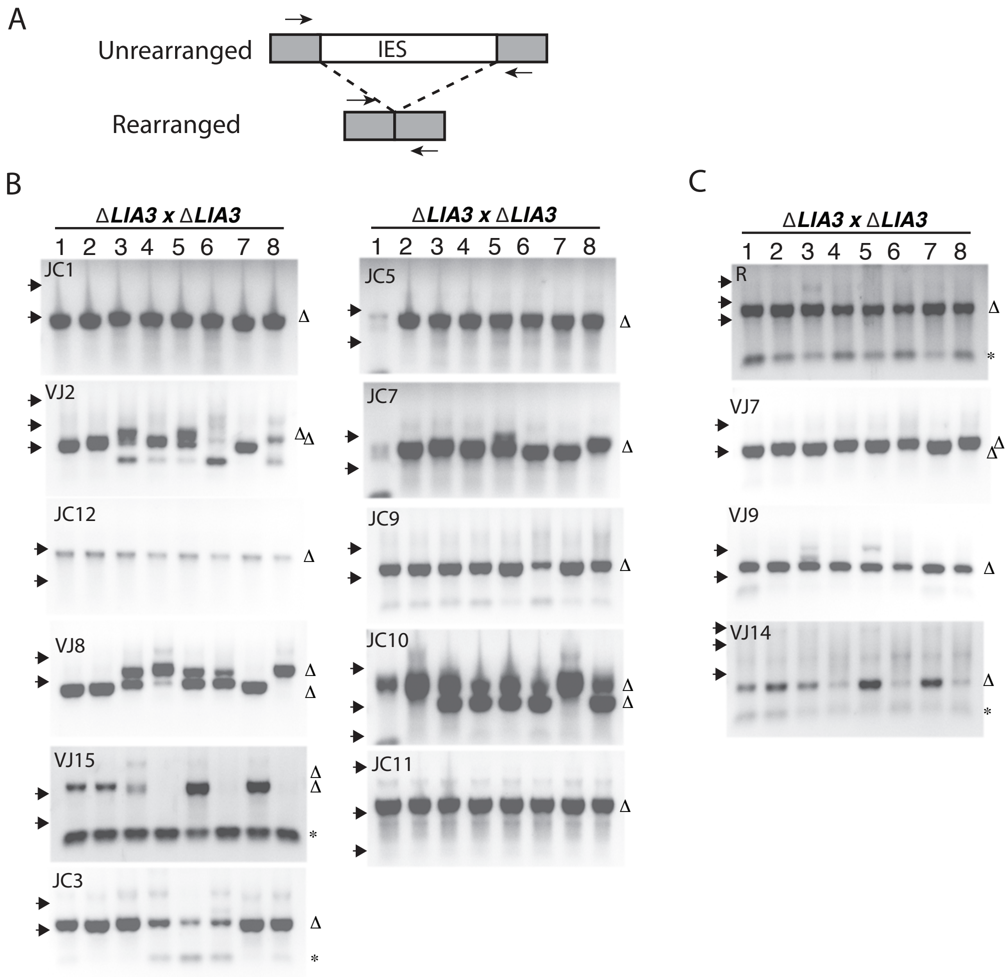

Supplementary Figure S5: IESs with excision defects in *ΔLTL1* progeny are unaffected in *ΔLIA3* progeny. A) Diagram depict the rearrangements of a generic IES locus whose normal rearrangements produce a single major form. Small arrows denote PCR primers flanking each IES used to amplify the loci from genomic DNA. B, C) PCR amplification of IESs in eight *ΔLIA3* progeny was used to determine the accuracy of IES excision: B) IESs largely unaffected in *Δ LTL1* progeny, or C) IESs that exhibit altered rearrangement in *ΔLTL1* progeny. Delta (Δ) symbols indicate the expected migration of products detected in wild-type strains (from analysis not shown). Solid arrowheads, the position of migration of size standards (bottom to top-250, 500,and 750bp). IES designation is given in left corner of each gel image.

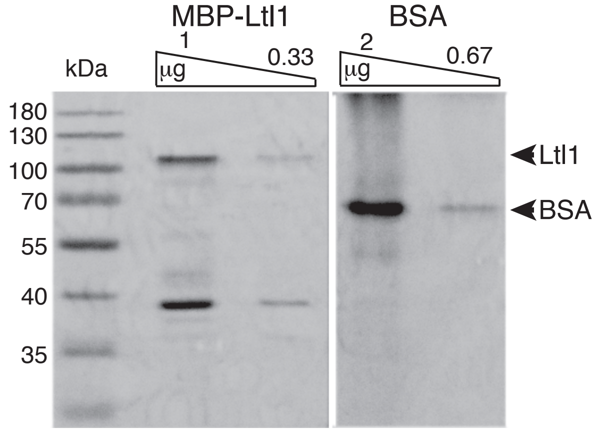

Supplementary Figure S6: Estimation of the concentration of Purified MBP-Ltl1. Purified proteins were visualized by coomassie-staining after electrophoresis of purified MBP-Ltl1 and known amounts (given above each lane) of Bovine Serum Albumin (BSA) on a SDS polyacrylamide. The amount of isolated full-length MBP-Ltl1 was estimated based comparison to the BSA standard. The sizes of pre-stained size markers are given on the left.

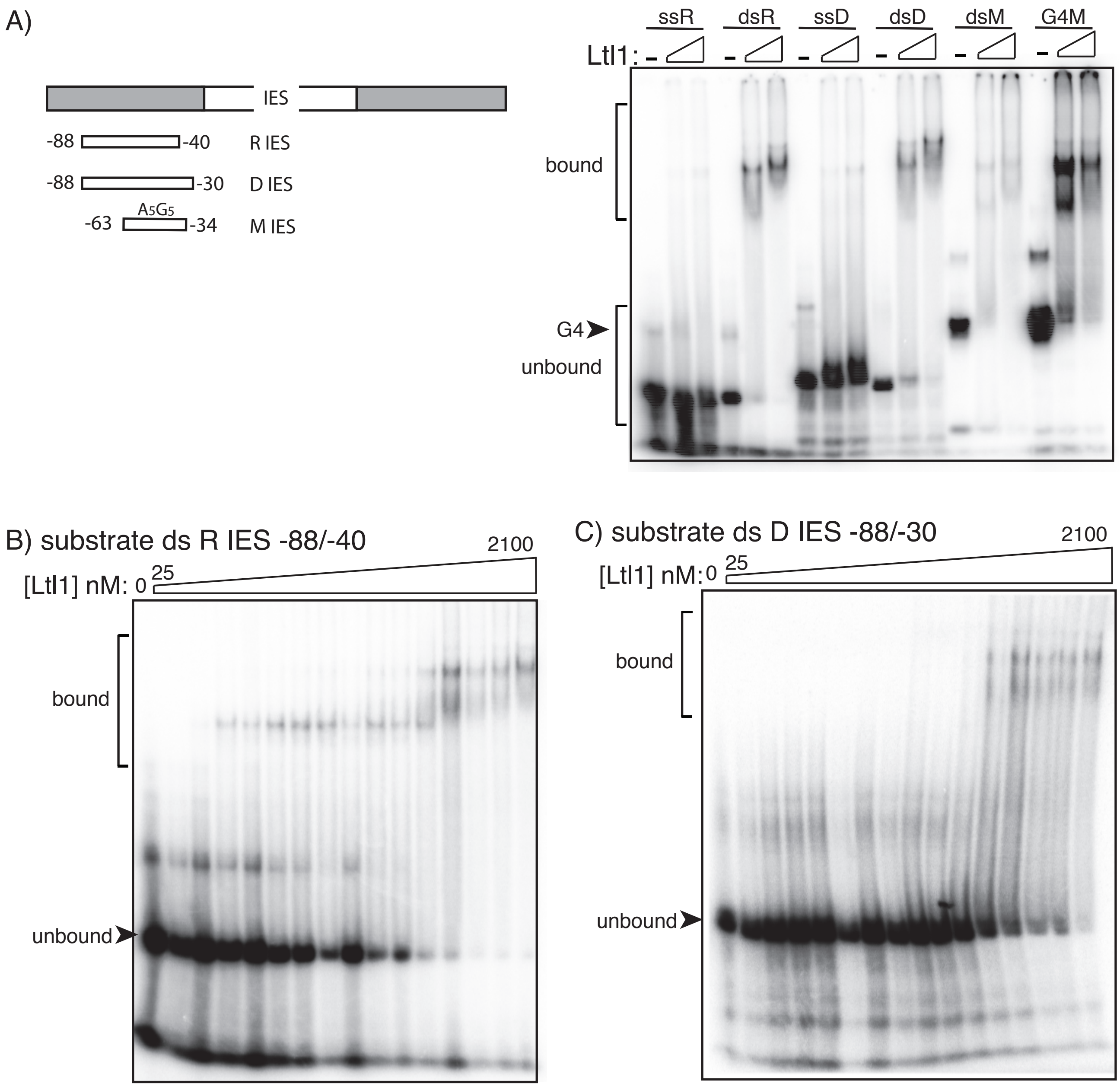

Supplementary Figure S7: Ltl1 binds to double-stranded DNA. EMSA using Ltl1 and radiolabeled substrates corresponding to regions flanking IESs. A) The diagram shows the position of binding substrates corresponding to sequences flanking the left side of the R, D, or M IES. B) EMSA using single-stranded R IES (ssR), double-stranded R IES (dsR), single-stranded D IES (ssD), double-stranded D IES (dsD), double-stranded M IES (dsM), and G-quadruplex (G4 M) for the M IES mixed with increasing amounts of MBP-Ltl1 (0, 1100, or 2100 nM) as indicated above each set of wells. The asterisk indicates that the majority of probe used in these reactions had formed a G-quadruplex. Brackets denote the migration of unbound and bound substrates. C) and D) EMSA shows binding upon titration with increasing amounts (0, 25, 50, 75, 100 150, 200, 50, 300, 400, 500, 700, 900, 1100, 1500, 2100nM) of MBP-Ltl1, which was used to determine Kd of Ltl1 binding to C) dsR(-88/40) and d) dsD(-88/30) IES left flanking DNA substrates.

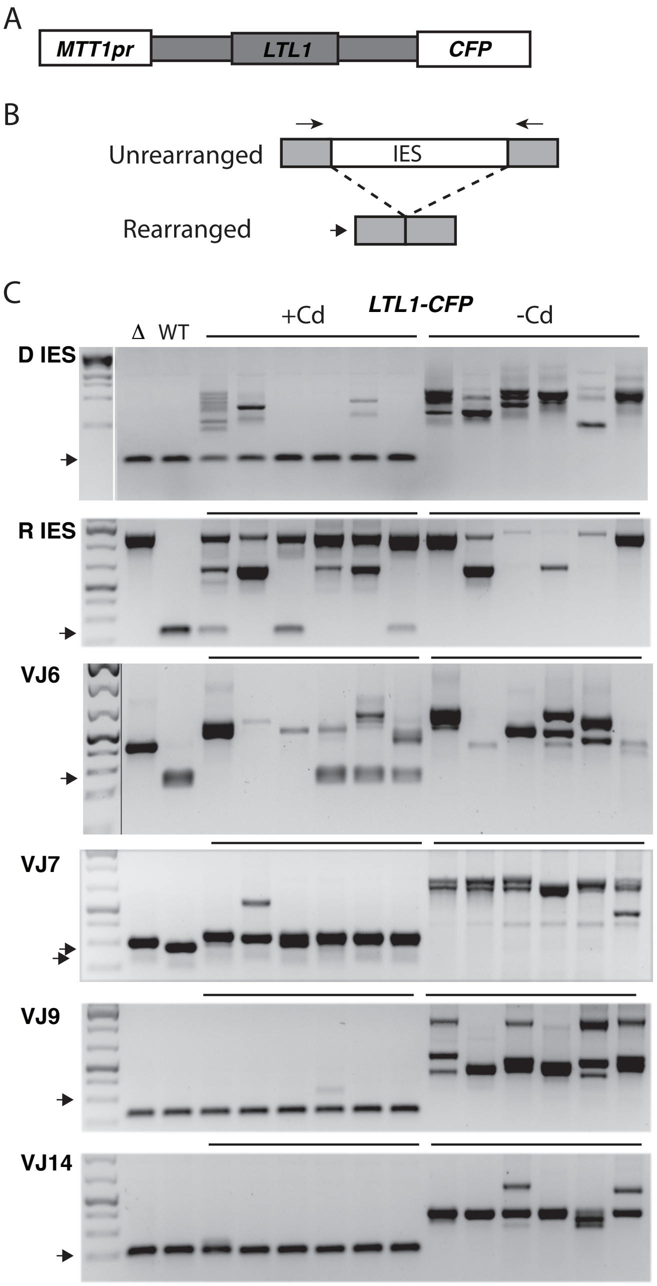

Supplementary Figure S8. Expression of Ltl1-CFP rescues IES excision defects in *Δ LTL1* progeny. A) The diagram indicates the intact *LTL1* coding region was fused to CFP and transformed into *ΔLTL1* cells. After expression from the cadmium (Cd)-inducible *MTT1* promoter the ability of the tagged protein to rescue excision defects was assessed by PCR. B) Diagram shows an general IES locus, which was monitored by PCR of genomic DNA isolated from the progeny of transformed cells with (+Cd) or without (-Cd) induced expression. Small arrows denote primers used to amplify the locus. C) Gel electrophoresis of PCR products that detect six IESs amplified from genomic DNA isolated from: a *ΔLTL1* (Δ) strain, a WT strain, or the progeny of *ΔLTL1* strains transformed with Ltl1-CFP. The arrowhead in B) and C) indicates the expected migration of PCR products corresponding to WT rearrangement products.
